# *Low Carbon Inducible2/Fatty Acid Desaturase4* locus in *C. reinhardtii* directs plastid peroxidase location and *trans* fatty acid production

**DOI:** 10.1093/plphys/kiaf394

**Published:** 2025-09-03

**Authors:** Timothy J Nicodemus, Stefan Schmollinger, John E Froehlich, Daniela Strenkert, Barbara B Sears, Christoph Benning

**Affiliations:** MSU-DOE Plant Research Laboratory, Michigan State University, East Lansing, MI 48824-6406, USA; MSU-DOE Plant Research Laboratory, Michigan State University, East Lansing, MI 48824-6406, USA; Department of Biochemistry and Molecular Biology, Michigan State University, East Lansing, MI 48824-6406, USA; MSU-DOE Plant Research Laboratory, Michigan State University, East Lansing, MI 48824-6406, USA; Department of Biochemistry and Molecular Biology, Michigan State University, East Lansing, MI 48824-6406, USA; MSU-DOE Plant Research Laboratory, Michigan State University, East Lansing, MI 48824-6406, USA; Department of Plant Biology, Plant Biology Laboratories, Michigan State University, 612 Wilson Road, Room 110, East Lansing, MI 48824-6406, USA; MSU-DOE Plant Research Laboratory, Michigan State University, East Lansing, MI 48824-6406, USA; Department of Plant Biology, Plant Biology Laboratories, Michigan State University, 612 Wilson Road, Room 110, East Lansing, MI 48824-6406, USA; MSU-DOE Plant Research Laboratory, Michigan State University, East Lansing, MI 48824-6406, USA; Department of Biochemistry and Molecular Biology, Michigan State University, East Lansing, MI 48824-6406, USA; Department of Plant Biology, Plant Biology Laboratories, Michigan State University, 612 Wilson Road, Room 110, East Lansing, MI 48824-6406, USA

## Abstract

Light capture and photosynthetic energy conversion depend on photosynthetic complexes that are embedded within lipid membranes. Components of these complexes are vulnerable to damage by reactive oxygen species, byproducts of photosynthesis that accumulate under environmental stress. Here we explore the basis for a lipid-based sensing mechanism allowing plants or algae to assess and respond to damage to the photosynthetic membranes. In *Chlamydomonas reinhardtii*, Low Carbon Inducible2 (LCI2) and Fatty Acid Desaturase4 (FAD4) are two proteins derived from the same locus by a differential splicing event, sharing an *N*-terminus encoded by the first two exons. FAD4 produces a 16-carbon, *trans* double bond-containing fatty acid found exclusively in phosphatidylglycerol of chloroplast membranes, while LCI2 recruits peroxidase activity to the membrane. The unique organization and transcriptional regulation of the *LCI2/FAD4* locus represents a regulatory interface that allows cells to initiate the biosynthesis of a fatty acid unique to the photosynthetic membranes while also linking it to the production of an enzyme involved in the mitigation of reactive oxygen species.

## Teaser

Mitigating stress-induced lipid damage in the chloroplast of *Chlamydomonas reinhardtii* involves the complex *Low Carbon Inducible2/Fatty Acid Desaturase4* genetic locus.

## Introduction

Lipid membranes separate living cells and ultimately organisms from their environment, and in the case of eukaryotes, allow for subcellular compartmentation, which is a cornerstone of cellular life. One such subcellular compartment is the chloroplast, an organelle common to all photosynthetic eukaryotic cells. Membrane integrity is essential for the buildup of a proton motive force driving ATP biosynthesis across specialized membranes such as thylakoids in chloroplasts or the inner mitochondrial membrane involved in photosynthesis or respiration, respectively (Mitchell 1961; Jagendorf and Uribe 1966). There is mounting evidence that the local lipid composition of the photosynthetic membrane has direct implications for the function and efficiency of resident membrane proteins (Kirchhoff 2018). Exposure of a photosynthetic organism to biotic and abiotic stresses can negatively affect the function and efficiency of energy-converting photosynthetic membranes, often stimulating the formation of reactive oxygen species (ROS) (Foyer and Hanke 2022; Hoh et al. 2023). Singlet oxygen is a highly reactive ROS formed under excessive light stress in photosystem II (Davis et al. 2016; Dmitrieva et al. 2020) that subsequently can give rise to different species of ROS, all of which lead in excessive amounts to oxidative damage of photosynthetic membranes (Choudhury et al. 2017). To circumvent photo-oxidative damage at the photosynthetic apparatus, antioxidative countermeasures are deployed by the cell including peroxidases and superoxide dismutases, both of which work to reduce excessive ROS pools and participate in reduction–oxidation (redox) signaling (Foyer and Hanke 2022; Hoh et al. 2023). Despite being vital for photosynthetic capacity and efficiency, relatively little is known about how the chloroplast detects ROS and signals damage to its photosynthetic membranes. Pigment–protein complexes involved in photosynthesis are embedded within the bioenergetic membrane and membrane lipids with their unsaturated fatty acid substituents may directly provide a ROS-sensitive substrate for oxidation, thereby generating potential oxylipin signaling molecules.

The lipid composition of the photosynthetic membrane is characterized by unique membrane lipids; one example is a particular molecular species of phosphatidylglycerol (PG) containing a 16:1*^Δ3trans^* (carbons: double bonds) acyl group. It is near ubiquitous within photosynthetic eukaryotes with one notable exception being orchids (Roughan 1986). Fatty Acid Desaturase 4 (FAD4) is the enzyme responsible for the production of 16:1*^Δ3trans^* in Arabidopsis (*Arabidopsis thaliana*) (Gao et al. 2009). When Arabidopsis FAD4 is lost there is no obvious change to the morphological phenotype under standard growth conditions (McCourt et al. 1985), although altered growth has been recently reported for the *fad4* mutant and *FAD4* overexpression lines under continuous light suggesting homeostasis related to this lipid is critical (Chen et al. 2023). Maintenance of photosynthetic performance despite chilling stress has been linked to the varying levels of 16:1*^Δ3trans^* in cow pea recombinant-inbred lines. The latter was corroborated in Arabidopsis *fad4* mutants and *FAD4* overexpression lines (Hoh et al. 2022). The activity of FAD4 is enhanced by peroxiredoxin PRXQ in Arabidopsis (Horn et al. 2020) linking the formation of 16:1*^Δ3trans^* to redox processes in the chloroplast. Furthermore, when the Arabidopsis FAD4- and PRXQ-encoding cDNAs were co-expressed in yeast, the formation of new fatty acids with a *Δ3cis* double bond was observed, but 16:1*^Δ3trans^* itself was not produced (Horn et al. 2020).

Structural analysis and modeling of the photosystem II complex (PS II) indicates the presence of PG, although PG containing 16:1*^Δ3trans^* was not explicitly investigated and its contribution to the binding of PG to PSII remains to be seen (Van Eerden et al. 2017). A classic study comparing thylakoids and photosynthetic complexes from the Arabidopsis *fad4* mutant and wild type suggested a role for PG containing 16:1*^Δ3trans^* in the oligomerization of light harvesting complex II, but PSII activity at different temperatures was not distinguishable (McCourt et al. 1985). Thus, it was generally concluded that 16:1*^Δ3trans^*-containing PG might play a role under specific conditions or may serve the fine-tuning of the activity of PSII. However, an essential mechanistic principle leading to the evolutionary conservation of 16:1*^Δ3trans^*-containing molecular species of PG in nearly all plants remains to be discovered.

Here we have taken the search for a specific function of 16:1*^Δ3trans^* in photosynthetic membranes to Chlamydomonas (*Chlamydomonas reinhardtii*), a unicellular green alga, whose last common ancestor with Arabidopsis and other land plants (*Viridiplantae*) diverged over a billion years ago (Merchant et al. 2007), yet 16:1*^Δ3trans^* is part of its thylakoid lipid profile (Giroud et al. 1988; Je et al. 2022). Analysis of the Chlamydomonas genome (Version 5.6) revealed a locus encoding a predicted ortholog of Arabidopsis FAD4, which was later annotated as a potential *FAD4* gene in version 6. Surprisingly, we discovered that the presumed *FAD4* locus overlaps with the locus for the *Low Carbon Induced 2* gene (*LCI2*), with both coding sequences appearing to be transcribed from the same promoter, sharing the same start codon and the first two exons.

Multiple genes are induced under carbon-limiting conditions in Chlamydomonas with many encoding components of the alga's carbon concentration mechanism (CCM). Their regulation appears to be under the control of the transcription factor CIA5 [previously also referred to as CCM1 (Fukuzawa et al. 2001; Xiang et al. 2001)] and they were originally referred to as “low CO_2_ inducible genes” or *LCI* including *LCI2* (Burow et al. 1996; Miura et al. 2004). Although the biochemical function of LCI2 remained unproven, early work noted that a stretch of the *LCI2* encoded protein had sequence similarity to ascorbate peroxidases (Moroney et al. 1998). These authors also observed that *LCI2* transcript abundance increases drastically under carbon-limited growth conditions and decreases to near undetectable levels when carbon is readily available. It should be noted that genes encoding components of the CCM are upregulated under conditions leading to oxidative stress (Choi et al. 2021; Neofotis et al. 2021). This suggests that some proteins participating in the low carbon induced activation of the CCM may also serve purposes of maintaining redox homeostasis.

One mechanism to protect against ROS damage involves proteins with peroxidase activity. A subclass composed of ascorbate peroxidases (APX) carries out the reduction of peroxide to two water molecules with ascorbic acid (AsA) as an electron donor resulting in one monodehydroascorbate (MDHA) molecule (Asada 1992). In plants, APXs are divided into four main categories, those that are membrane bound (mAPX), present in the chloroplast stroma (sAPX), the thylakoid membranes (tAPX), and in the cytosol (cAPX) (Shigeoka et al. 2002). The chloroplast ascorbate peroxidases tAPX and sAPX are known to be inactivated within seconds of depleting ascorbate while other forms such as mAPX and cAPX are far more stable and remain active longer providing a means to distinguish them in extracts (Nakano and Asada 1987). Ascorbate is regenerated through the oxidation of glutathione by dehydroascorbate reductase, which represents a node in multiple redox cascades and nuclear signaling (Dietz 2016). Reduction of APX activity results in increased oxidative damage when plants are challenged with abiotic oxidative stresses like ozone treatment, and *APX* genes are known to be induced under ROS-generating high light conditions (Örvar and Ellis 1997; Fryer et al. 2003).

In Arabidopsis, 16:1*^Δ3trans^* fatty acid biosynthesis is disrupted in the *fad4* mutant (Browse et al. 1985) while reconstitution of the biosynthetic pathway in yeast requires FAD4 and the stromal peroxiredoxin, PRXQ (Horn et al. 2020). As mentioned above, loss of 16:1*^Δ3trans^* has only subtle effects on plant growth or performance although it is nearly ubiquitous in terrestrial plants. To potentially reduce complexity and gain a fresh perspective on the molecular function of 16:1*^Δ3trans^* we turned to the model microalga Chlamydomonas. Hence, the present study examines the Chlamydomonas *LCI2/FAD4* locus and its expression encoding LCI2 and FAD4 with part of LCI2 at its N-terminus and proposes their biochemical roles.

## Results and discussion

### The complex *LCI2/FAD4* locus and its evolutionary origin

**Figure 1.**
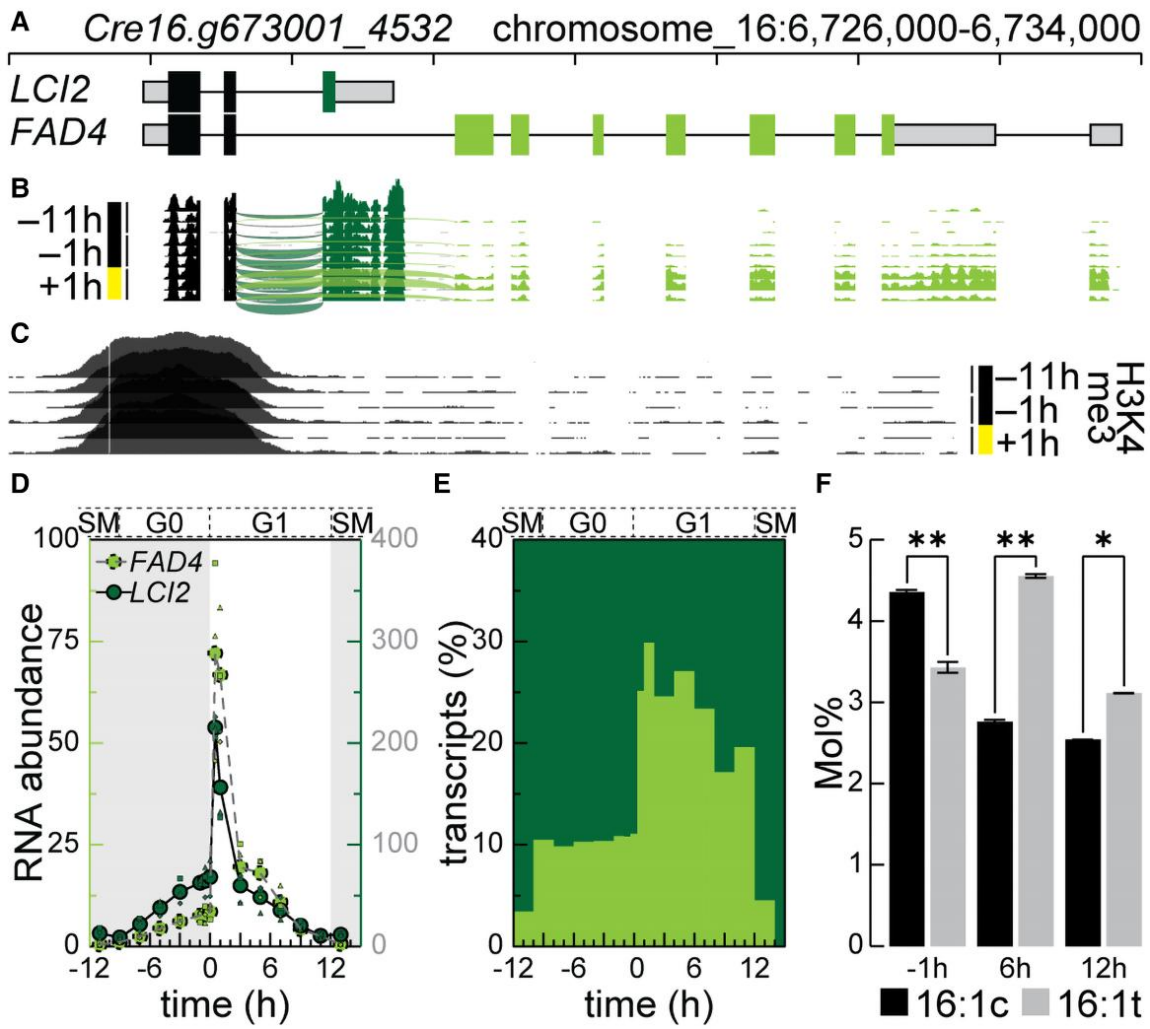
Locus *Cre16.g673001_4532* is alternatively spliced into two transcripts (*LCI2* and *FAD4*), which are differentially expressed throughout the day/night cycle (12 h/12 h). **A)** Two transcripts encoding for LCI2 and FAD4, respectively, originating from chromosome 16 (6,726,000 to 6,734,000) with common (black), LCI2-unique (dark shade of green), untranslated (gray) and FAD4-unique (light shade of green) exons (modified from transcripts *Cre16.g673001_4532.1–4,* Chlamydomonas genome v6.1 (Craig et al. 2023). **B)** Illumina short read coverage of the *FAD4/LCI2* locus in synchronous Chlamydomonas cell cultures, at time point −11 h and −1 h before (black) and +1 h after lights on (yellow, adapted from Strenkert et al. (2019)), including reads spanning intron 2 of *LCI2* (dark green, below) or *FAD4* (light shade of green, above). Shown are three independent replicates for each timepoint. **C)** Histone H3, lysine 4 trimethylation (H3K4me3) coverage, indicative of transcriptional start site, at the locus at the same points during a diurnal cycle (adapted from Strenkert et al. (2022)). Shown are two independent replicates for each timepoint. **D)** Transcript-specific expression of *FAD4* (light shade of green fill, left scale) and *LCI2* (dark shade of green fill, right scale) during the diurnal cycle. Shown is the number of fragments per kilobase of transcript per million reads mapped (FPKM, RNA-seq) of the average (large circle) and each of the three independent replicates (smaller triangle, square, diamond). **E)** Fraction of *FAD4* transcripts (light shade of green) and LCI2 transcripts (dark shade of green) among all transcripts originating from the locus (%) along the diurnal cycle. **F)** 16:1*^Δ9cis^* (16:1c, black) and 16:1*^Δ3trans^* (16:1t gray) abundance (mol%) during the diurnal cycle. Sd is indicated with *n* = 3. A two-tailed *t*-test was used. Significance is noted by an asterisk with *P*-values of >0.05 and a double asterisk for samples with a *P*-value of >0.01.

The Chlamydomonas genome encodes a protein with high sequence identity to functional domains, including four *trans*-membrane helices, of Arabidopsis FAD4, Cre16.g673001_4532 (v6.1) (Fig. 1). Notably, a previous analysis of the same locus prior to the discovery of FAD4 had identified a gene coding for a 131 aa protein, LCI2, that was described as being involved in CO_2_ homeostasis (Burow et al. 1996). Its protein sequence has similarity to membrane APXs.

To explore the evolutionary origin of LCI2, we aligned different stromal and thylakoid-associated APXs with LCI2 of the *Viridiplantae*, including various chlorophyte and streptophyte algae as well as a variety of monocot and dicot plants (Supplementary Fig. S1A, Supplementary Table S1). Soluble and thylakoid-associated APXs of all organisms have high sequence similarity, are structurally similar and differ mostly by the lack of a C-terminal extension found only in thylakoid-associated APX (Supplementary Figs. S1C and S2). Sequences similar to LCI2 were found in each genome, either as an individual gene in most chlorophyte and some streptophyte algae, or as a C-terminal extension in thylakoid APXs of land plants (e.g. TAPX in Arabidopsis, Supplementary Figs. S1B and S2). While most plants contain multiple proteins similar to the soluble and thylakoid-associated APX of Arabidopsis, chlorophyte and some streptophyte algae only contain APX sequences lacking the C-terminal extension. Interestingly, we found two *streptophypte* algae, *Spirogloea muscicola* and *Zygnema cf. cylindricum*, containing APX sequences that have a C-terminal extension similar to LCI2, which leads us to hypothesize that association of LCI2 and APX happened early in the streptophyte lineage.

### Differential splicing gives rise to LCI2 and FAD4

The sequences similar to *At*FAD4, including the fully conserved lipid desaturase domain (PF10520), are not part of LCI2, but are encoded by a second set of transcripts derived from the same locus (Fig. 1). Both transcripts include the first two exons, before an alternative splicing event of intron 2 produces either predominantly the shorter (990nt, 131aa) LCI2-encoding or less abundantly, the longer FAD4-encoding (2,471nt, 445aa) transcript. This association is not unique to Chlamydomonas. In several chlorophyte algae of the Chlorophyceae class, of which Chlamydomonas is a part of, the *LCI2* gene was found directly neighboring *FAD4* (Supplementary Fig. S1B), with a similar intron/exon structure, allowing for a similar mechanism to occur in this group of chlorophyte algae.

To confirm the alternative splicing event, we reanalyzed previously published data for the presence of both transcripts. We identified both transcripts in expressed sequence tags (EST) libraries of Chlamydomonas (Supplementary Fig. S3A), and most importantly, identified intron-spanning reads directly confirming both splice junctions (*LCI2* and *FAD4*) in Illumina RNA-seq short-read libraries from cultures grown phototrophically in a 12 h dark, 12 h light diurnal cycle (Fig. 1B, Supplementary Fig. S3B) (Strenkert et al. 2019). The presence of specific chromatin marks in specific genomic loci corresponds to differential expression of the underlying gene but can also be used to identify regulatory regions (Li et al. 2007). Trimethylated lysine 4 at histone H3 (H3K4me3) is a well-studied histone modification that specifically marks promoter regions of genes, independent of their expression levels, a feature that has been exploited to validate transcriptional start sites (TSSs) of previously undescribed genes like those encoding long noncoding RNAs (Strenkert et al. 2022). On the other hand, monomethylated lysine 4 at histone H3 (H3K4me1) is inversely correlated with trimethylated H3K4me3 and was depleted from gene promoters. To investigate whether *LCI2* and *FAD4* share the same TSS, we remapped previously published H3K4me1/3 ChIP-seq data (Chromatin ImmunoPrecipitation followed by deep sequencing) to the most recent version of the Chlamydomonas genome and analyzed the *LCI2/FAD4* locus (Strenkert et al. 2022; Craig et al. 2023) (Fig. 1). A single peak at the 5′ end of the *LCI2/FAD4* locus suggests that both genes share a single TSS, indicated by both a reduction of H3K4me1 and a corresponding increase of H3K4me3 (Fig. 1C; Supplementary Fig. S4). Since both transcripts appear to utilize the same promoter, we then analyzed if *LCI2-* and *FAD4-*specific transcripts accumulate differentially, using an experiment following cell cycle progression (Fig. 1, D and E, Supplementary Fig. S3C). Overall, the common first two exons and the third, *LCI2*-specific exon and 3′UTR showed higher expression compared to the downstream *FAD4*-specific exons and UTR throughout the day. Both transcripts peaked early in the day (0.5 to 1 h after lights-on), suggesting an increased demand for both proteins in the light, with varying contributions from *LCI2* and *FAD4*. Interestingly, while *LCI2* was produced at ∼10× the rate of *FAD4* during the night, the *LCI2:FAD4* ratio dropped to ∼4:1 during the day (Fig. 1, Supplementary Fig. S3). Early in the night, when cells undergo mitotic division, *LCI2* transcripts are produced at ∼20× the rate of *FAD4*, albeit at low amounts compared to the totality of transcripts produced during peak expression early in the day. The *FAD4*-specific transcript levels also correspond to the relative levels of 16:1*^Δ3trans^*, which also peaked in the light, but not 16:1*^Δ3cis^* fatty acids, which accumulate at the end of the night (Fig. 1F). Since *LCI2* was first identified in the context of CO_2_ limitation, we also examined earlier works on the relative expression of *LCI2* and *FAD4* in response to CO_2_ availability and in the absence of the carbon response regulator CIA5 (Supplementary Fig. S5, A and B; Brueggeman et al. 2012; Fang et al. 2012)*. LCI2* responded strongly with an increase in expression as the CO_2_ concentration decreased, while *FAD4* did not, shifting the balance more toward *LCI2* (Supplementary Fig. S5A). This is the opposite direction of what was observed early in the day (Fig. 1E), suggesting that the alternative splicing mechanism governing this locus (Fig. 1) responds to the availability of carbon, favoring LCI2 production early upon CO_2_ limitation. Interestingly, the fraction of *FAD4*-specific transcripts was increased in *cia5* mutants in all CO_2_ environments, up from ∼1 in six transcripts in control cultures to ∼1 in three in *cia5* mutants (Supplementary Fig. S5B), perhaps indicative of a role of CIA5 in balancing *LCI2/FAD4* transcript abundance, with an emphasis of favoring production of the CO_2_-responsive *LCI2* transcript.

### Disruption of the *LCI2/FAD4* locus does not affect photoautotrophic growth

**Figure 2.**
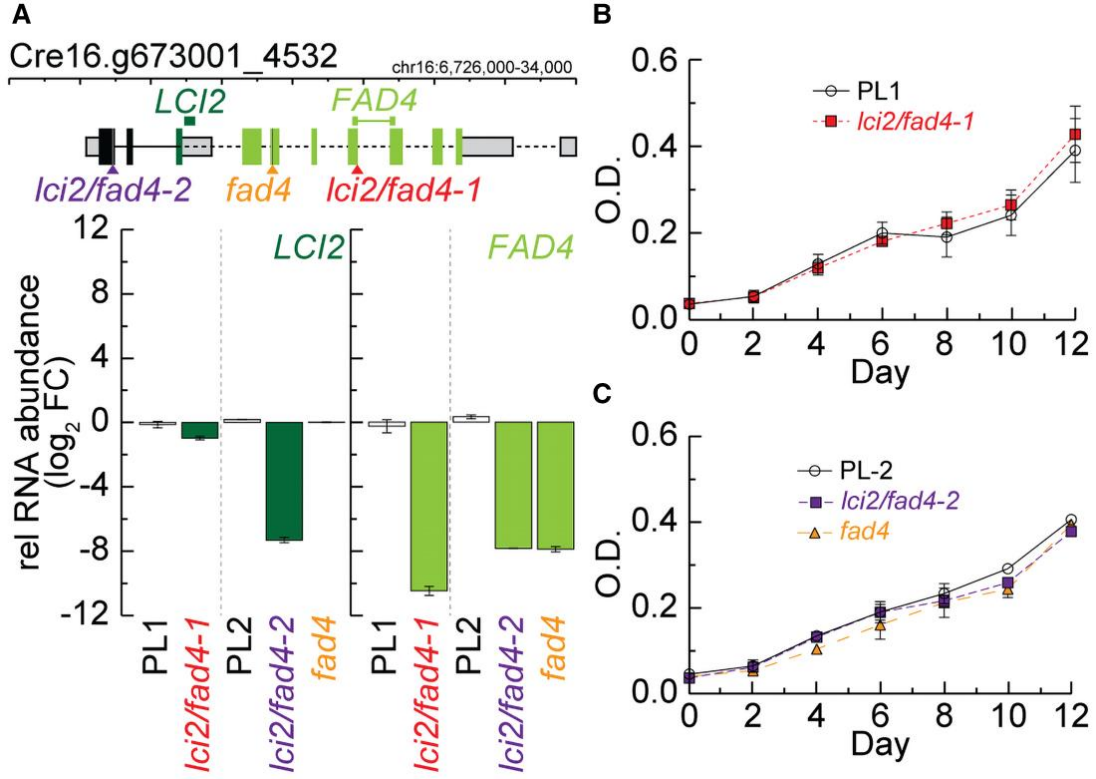
Disruption of *LCI2* and *FAD4* does not affect photoautotrophic growth. **A)** Structure of the *LCI2/FAD4* locus annotating the sites of disruption for each of the respective *lci2/fad4*, *fad4* mutants and the relative abundance of transcripts produced by CLiP (*lci2/fad4-*1) and CRISPR *lci2/fad4*, *fad4* mutants relative to their respective parental lines (PL-1 and PL-2, respectively). Colors are as defined in Fig. 1A. **(B)** and **(C)** show the growth of CLiP and CRISPR mutants relative to their respective parental lines over the course of 12 d in Tris Phosphate (TP) medium without supplemented carbon. Three biological replicates were averaged. Error bars represent Sd.

We applied a reverse genetics approach in combination with fatty acid profiling to dissect the function of LCI2 and FAD4 in fatty acid desaturation and to understand the functional relationship between the *LCI2* and *FAD4* genes that share the same promoter. We obtained an available *lci2/fad4* insertional mutant (Fig. 2A) from CLiP (Li et al. 2019) (*lci2/fad4-1*), which harbors an insertion in exon 6, disrupting *FAD4* expression. This insertional mutant showed no expression of *FAD4*-specifc transcripts, while *LCI2*-specific transcripts were reduced ∼50% (Fig. 2A). Therefore, we generated additional mutants using CRISPR/Cpf1-mediated gene editing based on single-stranded oligodeoxynucleotides (ssODNs) repair templates (Pham et al. 2022). We identified mutant strains with added early *in-frame* stop codons in exon 1, not producing both *LCI2* and *FAD4*, designated *lci2/fad4-2* (Fig. 2A), and strains specifically not producing *FAD4* (exon 4 stop codons), depicted *fad4* (Fig. 2A). We confirmed that neither *LCI2* nor *FAD4* were expressed in *lci2/fad4-2* strains by RT-qPCR, while only *FAD4* transcript abundance, but not *LCI2's*, was reduced in *fad4* (Fig. 2A).

To determine the effects of these mutations on photoautotrophic growth, optical density of cultures growing in minimal medium was measured daily over a 2-wk period as shown in Fig. 2, B and C. The *lci2/fad4-1* mutant did not display a growth phenotype when compared to the respective background strain (Fig. 2B). Similarly, *lci2/fad4-2* and *fad4* strains also did not exhibit a discernable difference in growth when compared to controls (Fig. 2C). Thus, disruption of the *LCI2/FAD4* locus is not detrimental to general phototrophic growth under the conditions tested.

### Disruption of the *LCI2/FAD4* locus causes specific fatty acid phenotypes

**Figure 3.**
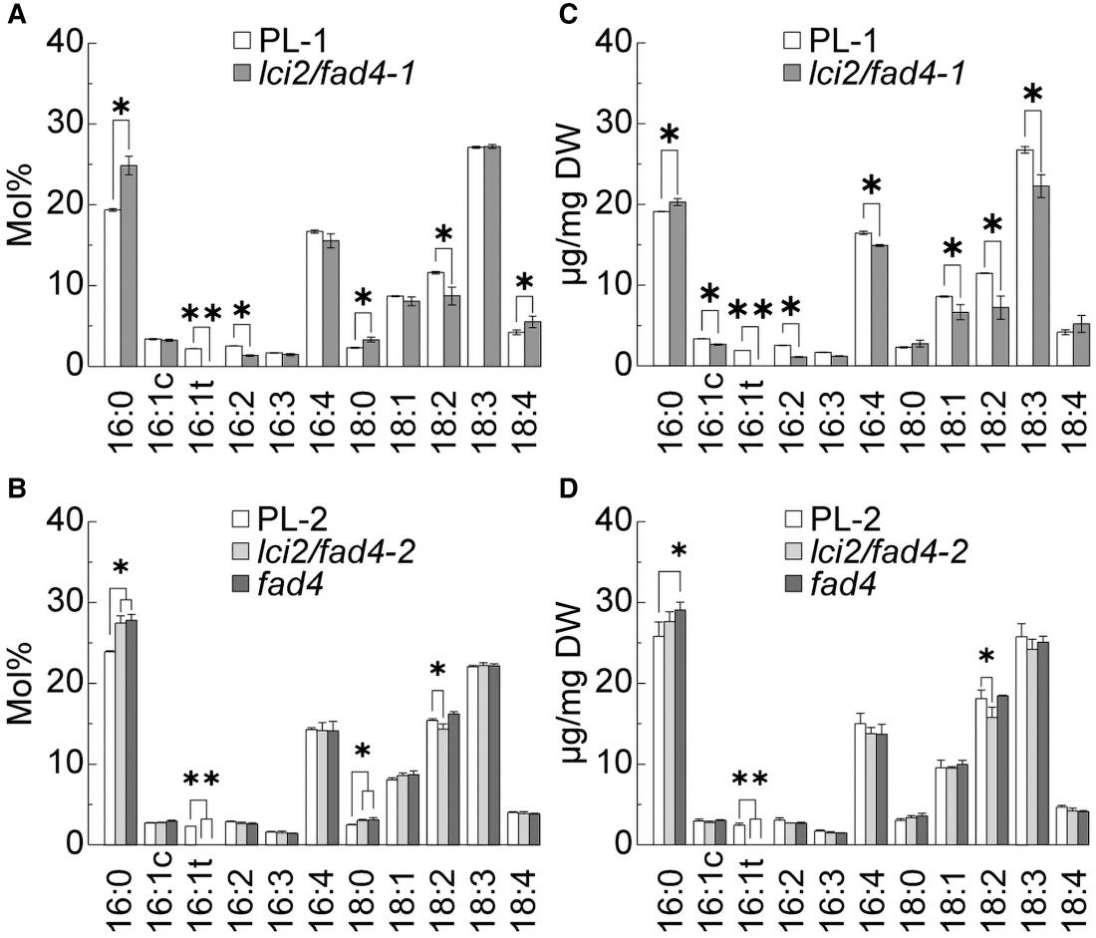
Fatty acid analysis of *lci2/fad4, fad4* mutants relative to their respective parental lines. **A)** Fatty acid composition of *lci2/fad4-1* mutant displayed as mol% and **B)** shown in *µ*g of fatty acid per mg of algal mass dry weight (DW) relative to its parental line PL-1. **C)** The fatty acid composition of *lci2/fad4-2* and *fad4* mutants displayed as mol% and **D)** shown in *µ*g of fatty acid per mg of algal mass DW relative to their parental line PL-2. Three biological replicates were averaged. Error bars represent Sd. One asterisk indicates *P* < 0.05 (two tailed *t*-test), two asterisks indicate *P* < 0.01.

Mutants affected in the expression of *FAD4* were grown mixotrophically and subjected to fatty acid methyl ester analysis following transmethylation of total lipids to probe for production of 16:1*^Δ3trans^* by the FAD4 desaturase (Fig. 3). Corroborating our hypothesis, 16:1*^Δ3trans^* was not present in any mutant with a disruption of the *LCI2/FAD4* locus leading to a loss or mutation of the FAD4-specific transcript (Fig. 3). More specifically, 16:1*^Δ3trans^* was not observed in the *fad4* mutant that expresses wild-type levels of *LCI2*, demonstrating that only the *FAD4* splicing product was responsible for the production of 16:1*^Δ3trans^* (Fig. 3, B and D). These data also showed that the *LCI2/FAD4* locus encodes the functional desaturase that is required to produce 16:1*^Δ3trans^*. Further examination of molar fatty acid ratios showed that all mutants harboring a loss of function mutation in the *LCI2/FAD4* locus contain increased 16:0 fatty acids, which is the precursor of 16:1*^Δ3trans^* (Fig. 3). Conversely, when examining the effects of *LCI2* disruption, a decrease in 18:2 fatty acid species is observed in all mutants with a disruption in *LCI2*, which was not observed in *fad4* mutants with intact *LCI2* (Fig. 3). These observations suggest that LCI2 also affects the fatty acid composition, but differently than FAD4.

### LCI2 affects APX activity

**Figure 4.**
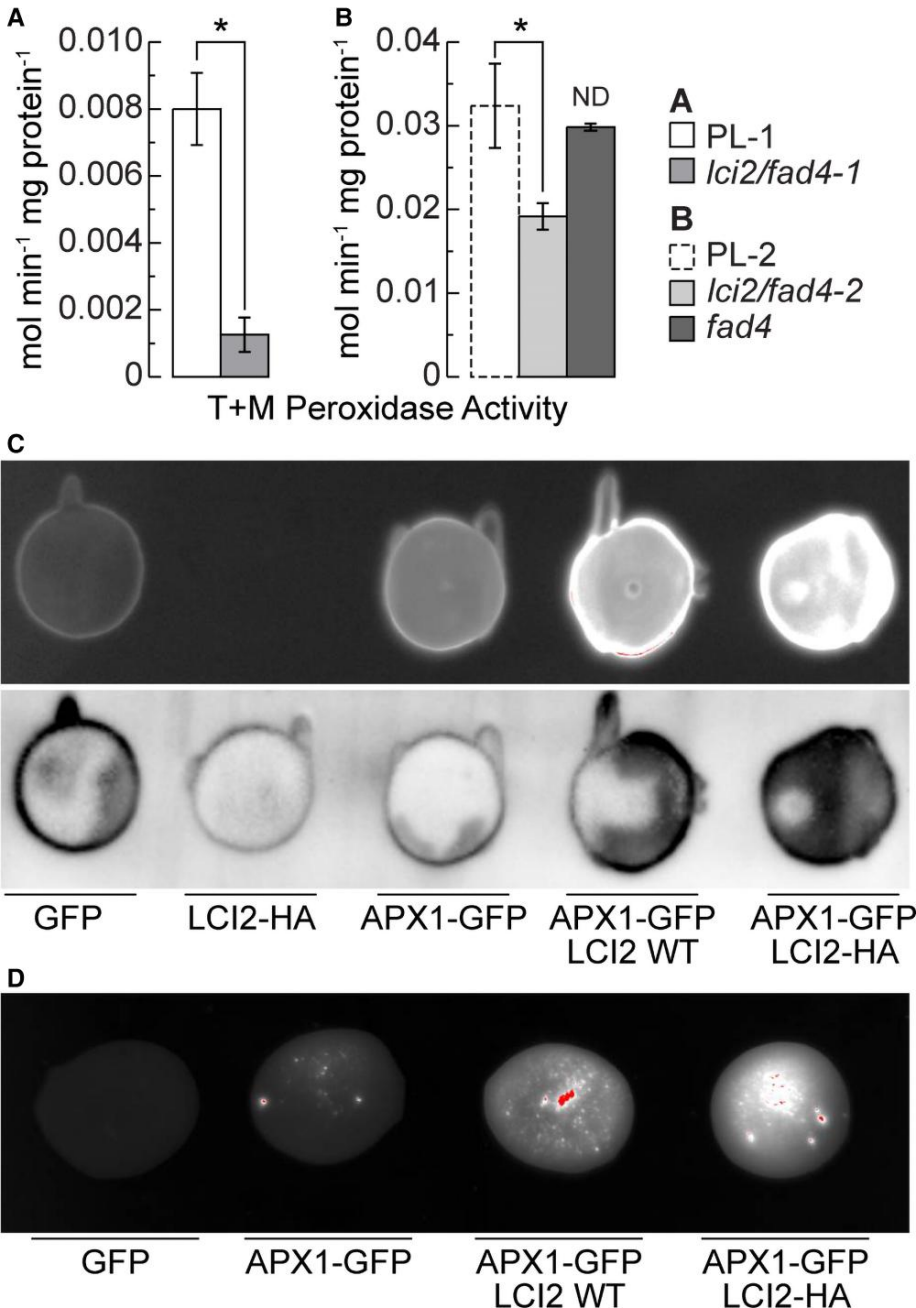
LCI2 stimulates membrane-bound ascorbate peroxidase activity. Peroxidase activity of pelleted cellular and thylakoid membrane fractions (T + M) of **(A)** parental line PL-1 and *lci2/fad4-1* and **(B)** parental line PL-2 and *lci2/fad4* and *fad4* mutants is shown. **C)**  *E. coli* (BL21) whole cell lysate fluorescence under UV (GFP, top) and Alexa 488 nm (HA tag, bottom) channels containing GFP, LCI2-HA, APX1–GFP, LCI2 WT + APX1–GFP, and LCI2-HA + APX1–GFP. **D)**  *E. coli* (BL21) purified membrane fraction fluorescence under UV excitation (GFP) following production of GFP, APX1–GFP, LCI2 WT + APX1–GFP, and LCI2-HA + APX1–GFP. **C, D)** the diameter for the spots is approximately 1 cm. Three biological replicates were averaged. Error bars represent Sd. One asterisk indicates *P* < 0.05 (two tailed *t*-test). ND, no significant difference.

As mentioned above, the *LCI2* coding region shows sequence identity with the C-termini of canonical plant thylakoid-bound APXs (Supplementary Fig. S1A). To determine whether LCI2 affects endogenous ascorbate activity, *lci2/fad4-1*, *lci2/fad4-2*, and *fad4* mutant cells were lysed and the extract fractionated by differential centrifugation to determine the relative ascorbate peroxidase activity of cytosolic/stromal and membrane bound peroxidases as shown in Fig. 4, A and B. The conversion of AsA to MDHA was measured spectroscopically and revealed a significant decrease in membrane peroxidase activity in both *lci2/fad4-1* and *lci2/fad4-2* double mutants as compared to their respective background strains (8- and 2-fold decrease, Fig. 4A). However, no decrease in membrane peroxidase activity was present in the *fad4* single mutant that still harbors functional LCI2, consistent with LCI2-dependent peroxidase activity in the membrane fraction (Fig. 4B). It should be noted that an increase in peroxidase activity was observed in cytosolic and stromal fractions of all *lci2* mutants (Supplementary Fig. S6, A and B). This result is consistent with a stromal compensatory response in mutants deficient in membrane thylakoid peroxidase activity or an increase in activity in stromal fractions due to failure to recruit peroxidase activity to the membrane. Alternatively, there may be a higher expression of the genes for cytosolic APX in all lines tested, which seems unlikely but cannot be ruled at this stage of the analysis.

### LCI2 affects APX1 membrane recruitment

Based on our current analysis we hypothesize that LCI2 serves as a membrane anchor recruiting the stromal APX1 in Chlamydomonas. In Chlamydomonas there are three APX isoforms: APX1 (Cre02.g087700.t1.2), APX2 (Cre06.g285150.t1.2), and APX4 (Cre05.g233900.t1.2). Of these, CrAPX1 and CrAPX2 show dual location in chloroplasts and mitochondria (Kuo et al. 2020) while CrAPX4 is a chloroplast enzyme (Caccamo et al. 2023). CrAPX2 has a twin-arginine transport (TAT) motif and is thus targeted to the thylakoid lumen. Likewise, CrAPX4 also appears to have a TAT targeting motif. Consequently, since CrAPX1 has a classical transit peptide, it is probably targeted to the chloroplast stroma and not the thylakoid lumen. Therefore, we focused our analysis on CrAPX1. To test this hypothesis, synthetic *LCI2* and *APX1* cDNA were inserted into the pDest14 overexpression vector and introduced into *Escherichia coli* BL21 cell lines. Dot blots of whole cell lysate expressing these genes were imaged using UV excitation and anti-hemagglutinin (HA) antibody chemiluminescence to visualize GFP fluorescence and HA tagged proteins respectively (Fig. 4C). It should be noted that there was significant overlap between the Alexa 488 nm channel used to image the HA chemiluminescent tag and the natural fluorescence of GFP. Presence of LCI2 was confirmed using alkaline phosphatase reaction (Supplementary Fig. S7). Co-expression of *LCI2* and *APX1–GFP* fusion proteins in BL21 lines was performed and membrane fractions were purified to determine if LCI2 was able to recruit APX1–GFP fluorescence to membrane fractions. Only a minimal amount of APX1–GFP fusion protein was present in membrane fractions when APX1–GFP was present by itself (Fig. 4D). However, when present with either native LCI2 or LCI2-HA the amount APX1–GFP fluorescence in purified membrane fractions was drastically increased (Fig. 4D). Taken together, these data are consistent with LCI2 recruiting stromal ascorbate peroxidase, APX1, to the membrane similar to plant thylakoid bound ascorbate peroxidases of seed plants that have a C-terminal membrane anchor.

### Loss of LCI2/FAD4 affects mixotrophic growth in the presence of 3-(3,4-dichlorophenyl)-1,1-dimethylurea

**Figure 5.**
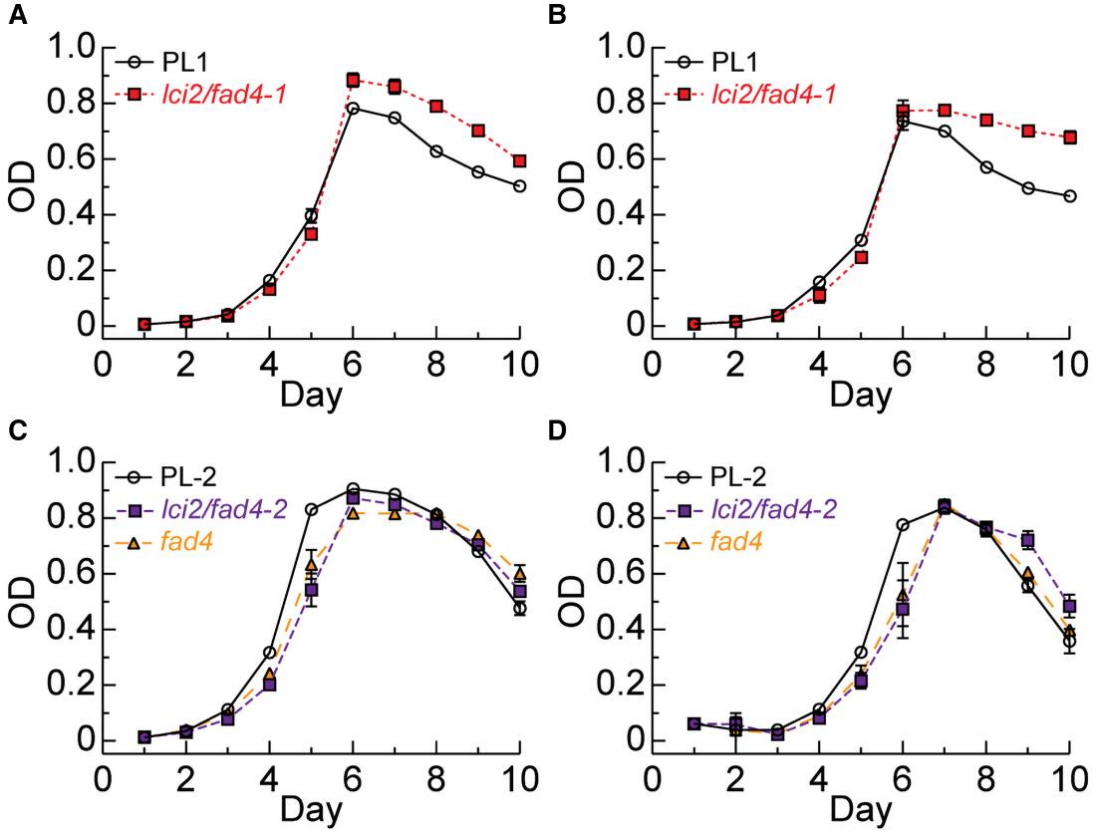
Growth and DCMU sensitivity of the different mutant lines. Growth (optical density of the culture, OD) of CLiP *lci2/fad4-1*  **(A, B)** and CRISPR *lci2/fad4* and *fad4*  **(C, D)** mutants relative to their respective parental lines (PL-1 and PL-2) in TAP medium in the presence of 1 *µ*m  **(A, C)** or 5 *µ*m  **(B, D)** DCMU (added on day 3). Error bars represent Sd (not shown when smaller than the symbols), *n* = 3.

To test how the loss of LCI2/FAD4 might affect redox-dependent processes, we determined the impact of partial and total photosystem inhibition with subsequent ROS production on mixotrophic growth following addition of 1 or 5 *μ*mol 3-(3,4-dichlorophenyl)-1,1-dimethylurea (DCMU, Roberts et al. 2003) at day 3 of the cultures (Fig. 5). The CRISPR lines *fad4* and *lci2/fad4-2* showed reduced growth until they reached the stationary phase (Fig. 5, C and D). The reduced function mutant *lci2/fad4-1* showed no or very mild growth impairment prior to reaching the stationary phase but reached higher levels of apparent cells during the stationary phase (Fig. 5, A and B), which was not as obvious for the CRISPR lines (Fig. 5, C and D). Because growth was measured by optical density, it is possible the mutants produced optical dense material during the stationary phase unrelated to growth. In any case, during exponential mixotrophic growth in the presence of DCMU, when ROS is produced the loss of LCI2 or FAD4 led to a reduction in growth suggesting that these proteins are involved in ROS mitigation when the electron transport chain comes under stress producing more ROS, in this case through addition of the herbicide DCMU.

### Loss of LCI2/FAD4 increases lipid oxidation

**Figure 6.**
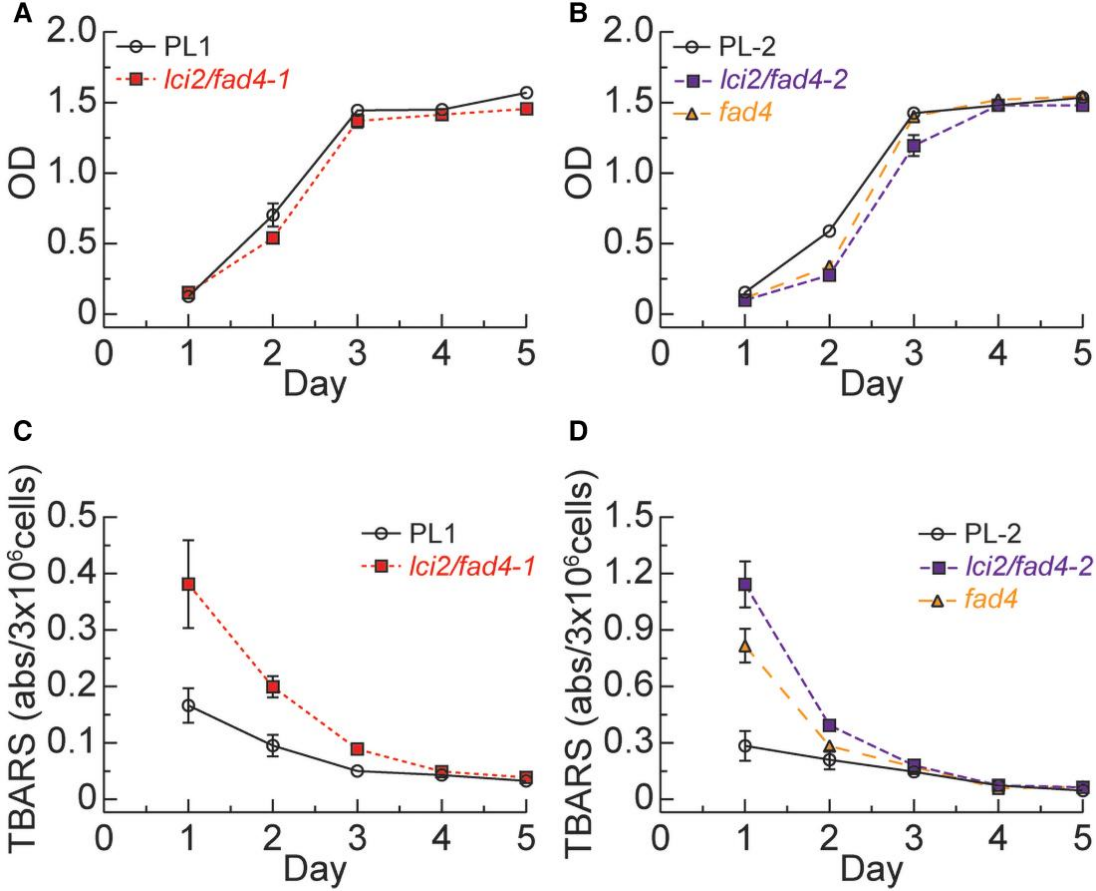
Growth and lipid oxidation in the different mutant lines. Growth **(A, B)** of *lci2/fad4-1*, *fad4*, and *lci2/fad4-2* and their respective parental lines in TAP medium over the course of 5 d during a lipid oxidation product (TBARs) analysis shown in **(C)** and **(D)**. Depicted is the absorbance of TBAR substrate normalized to 1 OD/mL or 3 × 10^6^ cells per mL. Error bars represent Sd (not shown when smaller than the symbols); *n* = 3.

To test whether the effect on growth of the *lci2/fad4* mutants is indeed related to loss of ROS protection we examined the amount of lipid oxidation. When grown mixotrophically, slight reductions in growth were observed for *lci2/fad4-1*, *fad4*, and *lci2/fad4-2* at various points in the growth curve relative to the parental lines (Fig. 6, A and B). When analyzing the amount of lipid peroxidation, all mutants lacking *FAD4* exhibited more than 2-fold the amount of lipid peroxidation compared to their respective parental lines with *lci2/fad4-2* showing nearly a 3-fold increase (Fig. 6, C and D). While *fad4* mutants also displayed more lipid peroxidation than the respective parental line, the observed increase was not as pronounced as in the *lci2/fad4-2* double mutant (Fig. 6D).

## Conclusion

**Figure 7.**
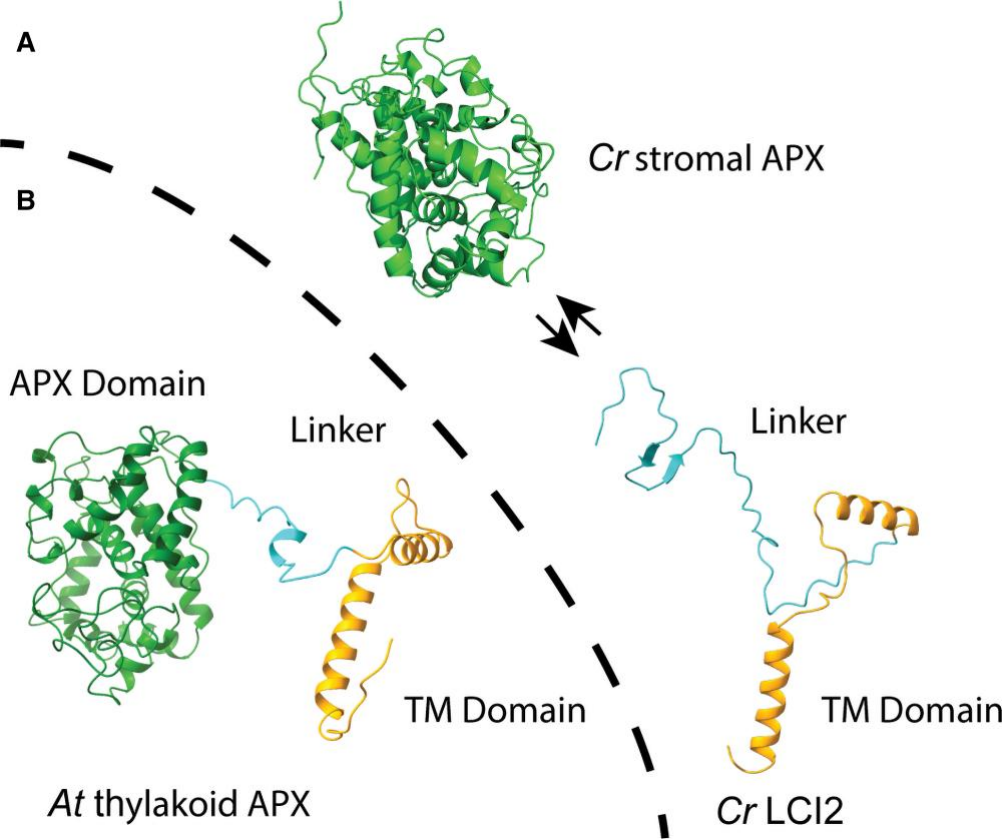
Hypothesized membrane recruitment of Chlamydomonas CrAPX1 by LCI2. AlphaFold 3.0 structure predictions for stromal CrAPX1 with membrane bound CrLCI2 **(A)** and **(B)**, thylakoid membrane-located Arabidopsis Ascorbate Peroxidase (AtAPX1); At1g77490). The Ascorbate Peroxidase Domain is shown in green; Linker region in Cyan and Transmembrane (TM) Domain in gold. All structures were generated by AlphaFold 3.0 (Abramson et al. 2024) and illustrated using ChimeraX (Meng et al. 2023; Pettersen et al. 2021).

In conclusion, while the detailed implications of co-expressing *LCI2* and *FAD4* in Chlamydomonas are still unknown, the work presented here strongly supports the assertion that the coregulation of FAD4 and LCI2, two proteins responsible for specialized lipid metabolism of thylakoid fatty acids and redox biochemistry, respectively, has functional relevance. This conclusion is supported by the fact that the structure of the *LCI2/FAD4* locus is not a peculiarity of Chlamydomonas alone but is conserved in other green algae (Supplementary Fig. S1). Lipids, specifically those containing double bonds, are susceptible to damage by ROS resulting in lipid peroxidation. In thylakoid membranes, lipids are found in close proximity to the photosynthetic complexes that continuously perform oxygenic photosynthesis. Therefore, it is reasonable to assume that lipids regularly encounter ROS formed during oxygenic photosynthesis, but more so under adverse conditions, which could potentially result in lipid-derived, mobile signaling molecules. This would allow cells to sense ROS damage to membranes since oxidized acyl groups are replaced through the de novo synthesis of new acyl chains or the remodeling of existing lipids. Repairing damaged lipids (acyl hydroperoxides) would require the activity of a lipid desaturase, FAD4, and ROS mitigating proteins, such as APX1 which we hypothesize based on the current analysis is recruited to the membrane by the membrane anchor LCI2 (Fig. 7). The change in redox state in the process and interaction of redox active proteins like APX1 and peroxiredoxin Q with other protein clients could affect directly or indirectly the activity of components of the photosynthetic apparatus, thereby providing a mechanism for feedback regulation in response to adverse conditions. One reason why LCI2 and FAD4 in Chlamydomonas might share the same N-terminus encoding a membrane anchor could be that they can interact through this anchor leading to the recruitment of APX1 to the vicinity of FAD4. The same may happen in plants where thylakoid APXs come with their own membrane anchor. This hypothesis and how APX1 and FAD4 interact biochemically in the production or turnover of 16:1*^Δ3trans^* and the possible production of lipid derived signaling molecules needs to be further explored.

## Materials and methods

### Strains and growth conditions

Chlamydomonas cultures were grown with constant agitation at a rate of 160 RPM at 25 °C in 12 h dark:12 h light cycles at 168 *μ*mol/m^2^/s or 24 h continuous light under Sylvania cool white lamps on TAP or TP medium as indicated (Harris et al. 2009). For maintenance, agar-solidified plates (1.5%) were grown on shelves with 170 *μ*mol/m^2^/s at room temp (25 °C). The *lci2/fad4-1* CLiP mutant (Li et al. 2019) was obtained through the Chlamy Library Project (https://www.chlamylibrary.org/) and back-crossed twice against the parental line CC4533 designated PL-1. Mutants generated using CRISPR gene editing were generated in the CC425 parental line designated PL-2.

### Locus analyses

The region on chromosome 16 of the Chlamydomonas genome (v5.6 https://phytozome-next.jgi.doe.gov/info/Creinhardtii_v5_6 and v6.1 https://phytozome-next.jgi.doe.gov/info/CreinhardtiiCC_4532_v6_1), encoding LCI2/FAD4, was analyzed for transcript production and structural features, using available data on phytozome (Goodstein et al. 2012) and from several previously analyzed genome-wide studies. The presence of trimethylated lysine 4 of histone H3 (H3K4me3) and absence of monomethylated lysine 4 of histone H3 (H3K4me1), indicative for TSSs, was analyzed across the whole *LCI2/FAD4* locus in samples acquired by chromatin-immunoprecipitation followed by deep-sequencing (ChIP-seq) from synchronized cells at the beginning of the night (−11 h dark), at the end of the night (−1 h dark) and early in the day (1 h light) (Strenkert et al. 2022). Short reads from this study were obtained from NCBI Short Read Archive (SRA) and aligned to the Chlamydomonas v6.1 genome (Craig et al. 2023) using the Burrows–Wheeler Alignment tool (Li and Durbin 2009). Alternative splicing events resulting in full-length *FAD4* and *LCI2*-specific transcript production from the locus were identified in aligned EST libraries (Asamizu et al. 1999, 2004; Goodstein et al. 2012), accessed through phytozome. Short reads RNA-seq samples generated from synchronized cells grown phototrophically in a 12 h dark:12 h light cycle (Strenkert et al. 2019), cells transitioning from 5% CO_2_ to 100 ppm CO_2_ (Brueggeman et al. 2012), and in wild-type and *cia5* mutant cells acclimated to various CO_2_ environments (Fang et al. 2012) were obtained from SRA, mapped using STAR aligner (Dobin et al. 2013) to the v6.1 genome, and analyzed for expression using Cufflinks (Trapnell et al. 2010). Expression of *LCI2-* (similar to model v5.6, Cre16.g673001.t2.1) and *FAD4*-specific transcripts (similar to model v5.6, Cre16.g673001.t1.1) was obtained using a modified GTF file that uses only these two transcript models at the *LCI2/FAD4* locus in the v6.1 genome. Reads spanning the second intron of either *LCI2* or *FAD4*, respectively, were obtained using samtools (Danecek et al. 2021) filtering. Figures were produced using phytozome (https://phytozome-next.jgi.doe.gov/), IGV (v2.14) (Robinson et al. 2011), and Adobe Illustrator.

### CRISPR mutant generation

Generation of *fad4* and *fad4/lci2-2* knock-out strains was facilitated using CRISPR/Cpf1-mediated gene editing at the *LCI2/FAD4* locus and was performed as described (Ferenczi et al. 2017; Greiner et al. 2017; Pham et al. 2022) . In brief, CC425, a cell wall reduced arginine auxotrophic strain, was used for transformation with an ribonucleoprotein (RNP) complex consisting of gRNAs targeting a PAM (protospacer adjacent motif) sequence in exon 4 of *FAD4* or exon1 within the *LCI2/FAD4* locus and LbCpf1 as shown in Fig. 1. Cells were grown to a density of 2 × 10^6^ cells per mL and counted using a Coulter counter Z2. Exactly 2 × 10^7^ cells were collected by centrifugation (3 min, 1,500 × *g*) and resuspended once in Maxx Efficiency Transformation Reagent (1 mL; ThermoFisher), followed by resuspension in 230 *μ*L of the same reagent supplemented with sucrose (40 mm). Cells were incubated at 40 °C for 20 min. Purified LbCpf1 (80 *μ*m) was preincubated with the two respective gRNAs (2 nmol, targeting exon 4 in *FAD4*: TTTGCGGAAGCCCTCAACAAGGTGG, targeting exon 1 in *LCI2*: TTTATGTGAACGCCGACGGACCCCG) at 25 °C for 20 min to form RNP complexes. For transfection, 230 *μ*L cell culture was supplemented with sucrose (40 mm) and mixed with preincubated RNPs, pCB412 plasmid (which contains the *ARG7* gene and complements the defective *arg7* gene in strain CC425 and thus confers the ability to grow without arginine supplementation in the medium). In order to achieve template DNA-mediated editing, the following ssODN were used: at the *FAD4* locus (4 nmol, sequence containing two in-frame stop codons after the PAM target site) (CACTCCCGAGATGCGCGCCTGGACTTGGGTGTCCATCTCCATGATGGGAGCCACCTTTGCGGAATAACTCTAGAAGGTGGGGGGGGGGCGGAGGCGGGGAGGGGGGAGCGGGGGGGGGGGAGAGGGGGCTGCAT) and at the *LCI2* locus (4 nmol, sequence containing two in-frame stop codons after the PAM target site (GTCACCGCGCCCGGCGCTTCGCAGACCACTCGTCGGGAGTCTGATGGCCTTTATTAAAACTAGGACGGACCCCGCCCGGTAAGTTATTTGCGCGGCGACCGAATGTGGGGCCCAGCTCAA). The final volume of the transformation reaction was 280 *μ*L. Cells were electroporated in a 4-mm gap cuvette (Bio-Rad) at 600 V, 50 *μ*F, 200 Ω by using Gene Pulser Xcell (Bio-Rad). Immediately after electroporation cells were allowed to recover overnight in darkness without shaking in 5 mL TAP with 40 mm sucrose and 0.4% (w/v) polyethylene glycol 8000 and then plated on TAP agar plates after collection by centrifugation (5 min at RT and 1,650 × *g*) using the starch embedding method (with 60% corn starch). After 12 d, colonies were transferred to new plates, grown for another 7 d and screened by colony PCR. The following program was used for all colony qPCRs: 95 °C for 5 min followed by 40 cycles of 95 °C for 15 s, and 65 °C for 60 s. To identify gene editing at the *FAD4* locus, oligos used for colony PCR were FAD4screenfor: AATAACTCTAGAAGGTGGGG and FAD4screenrev: GCTACTCAAGCCAAGCCCCT; at the *LCI2* gene locus, the oligos were FAD4LCI2screenfor: GATGGCCTTTATTAAAACTAG and FAD4LCI2screenrev: GTTTGGAAGTAAGAGGCGGCC. PCR products identified candidates likely to have been successfully edited at the respective loci due to annealing of the primers to the altered sequence. Genomic DNA was extracted from one clone for each target that showed successful qPCR amplification (either at the *LCI2* or the *FAD4* locus) and sequenced by Illumina sequencing for genotyping. For *LCI2* ssODN-mediated gene editing introduced two in-frame stop codons within the first exon. For the *FAD4* gene, editing introduced two in-frame stop codons within exon 4 of *FAD4* (Fig. 1).

### RT-qPCR

To isolate RNA and perform real time quantitative PCR (RT-qPCR), approximately 2 to 5 × 10^7^ cells were collected by centrifugation for 5 min at 1,424 × *g*, 4 °C. RNA was extracted using Trizol reagent. The Zymo Research RNA Clean & Concentrator-5 Kit including DNAse I digest was used according to the manufacturer’s instructions. Reverse transcription was primed with oligo dT(18) using 2.5 *µ*g of total RNA and SuperScript III Reverse Transcriptase (Invitrogen) according to the manufacturer's instructions. The subsequent cDNA was diluted 15-fold before use. RT-qPCR reactions contained 5 *μ*L of cDNA corresponding to 200 ng of total RNA, 6 pmol of each forward and reverse oligonucleotide, and 10 *μ*L of ITAQ Mastermix in a 20 *μ*L volume. The following program was used for all RT-qPCRs: 95 °C for 5 min followed by 40 cycles of 95 °C for 15 s, and 65 °C for 60 s. Fluorescence was measured at the end of each 65 °C cycle. A melting curve analysis was performed at the conclusion of the cycles from 65 to 95 °C with fluorescence reads every 0.5 °C. *Rack1* has been described previously and served as reference transcript. The following oligonucleotides were designed to amplify *LCI2* or *FAD4*, respectively: LCI2For CTGGGGCTGCGGTTTTGTGG, LCI2Rev TGCGCTGGTGTCACGAAGGA, FAD4For GCCAGATCGCCGCATTCCAG, FAD4Rev GCGTCCCAGCCCACAGACAT (Shown in Fig. 1).

### Lipid extraction

Lipid analysis was carried out on total lipid extracts of algal cells, lyophilized for 24 h prior to lipid extraction. Cell pellets were subjected to mechanical disruption via bead milling at a frequency of 30 Hz for 3 min. Lipid extraction was carried out according to Bligh and Dyer (1959) with modifications. In brief, disrupted cells were resuspended in extraction solution 20:10:1 (v/v/v) chloroform:methanol:formic acid. The mixture was vortexed for 30 s and then tubes were spun at 13,000 × *g* for 1 min. The supernatant was removed and transferred to glass tubes and the process was repeated once. One half volume of methanol containing 0.2 m phosphoric acid and 1 m KCl was added to glass tubes. Tubes were spun at 3,000 × *g* for 10 min, 4 °C resulting in a clear upper phase, an opaque interphase, and a green lower phase. The lower phase was removed with care not to disturb the interphase and transferred to a clean glass tube and stored at −20 °C until analysis.

### Lipid analyses

Lipids were converted to Fatty Acid Methyl Esters (FAMEs) by transmethylesterification. Lipid extracts were evaporated under nitrogen until dry prior to addition of 3 mL of 1 N HCl in methanol and sealing of the tubes. Samples were placed in a water bath at 80 °C for 25 min. After removal and cooling to room temperature, 0.2 mL of hexane and 2 mL of saturated NaCl solution were added to each tube. The samples were then spun at 3,000 × *g* in a tabletop centrifuge for 10 min. The hexane layer was then removed and transferred to a GC sample vial and sealed. FAMEs were analyzed by Gas Chromatography Flame Ionization Detection (GCFID) using an Agilent 7890A gas chromatograph equipped with FID detection. A description of GCFID parameters is as follows. Samples were analyzed with a 1:10 split ratio, inlet pressure was set to 18 PSI with a total flow rate of 29.5 mL/min and a 1 mL/min purge flow rate. The initial oven temperature was set to 130 °C and held for 1 min, increased by 6.5 °C/min to a temperature of 170 °C, then increased by 8 °C/min to 194 °C, and a final ramp of 20 °C/min to 230 °C. Detector parameters were as follows: the detector temperature was set to 300 °C with an H_2_ flow of 30 mL/min and an air flow of 400 mL/min.

### Molecular biology

Protein sequences were retrieved from Phytozome (v13) using the *C. reinhardtii* v5.6 database for the following: Cre02.g087700.t1.2 (CrAPX1: L-ascorbate peroxidase) and Cre16.g673001.t1.1(CrLCI2: Low-CO_2_ inducible protein). Subsequently, the following constructs: *LCI2-HA*, *GFP-CrAPX1*, *GFPCrAPX1/CrLCI2HA*, and *GFPCrAPX1/CrLCI2* were synthesized and inserted into the vector pENTR-SD-DTOPO (Invitrogen) (TWIST Bioscience, San Francisco, USA). Finally, using a Clonase II reaction assay as describe by the manufacturer protocol (Invitrogen), *LCI2-HA, GFP-CrAPX1*, *GFPCrAPX1/CrLCI2HA*, and *GFPCrAPX1/CrLCI2-WT* were inserted into pDEST14 (Invitrogen).

### Structural biology

AlphaFold 3.0 (Abramson et al. 2024) structure predictions of various thylakoid APX from different plants and algae was performed using protein sequences that were obtained from Phytozome (v13, March 1, 2024; Goodstein et al. 2012). Subsequently, for all models, the quality of structural models predicted was characterized by the mean of per residue pLDDT score (predicted Local Distance Difference Test). For each AlphaFold 3.0 run, 5 models were generated and ranked by score (0 to 4 best to lowest confidence). Accordingly, for our structural analysis, the best rank model was used for our comparative analysis. All models were illustrated using PyMOL2 (The PyMOL Molecular Graphics System. http://www.pymol.org).

### Peroxidase assays

APX assays were performed in a manner consistent with analysis of plant vegetative tissue modified according to Yoshimura et al. (2000). Algal cells were grown on TAP medium containing agar plates for 4 d in 12 h light cycles at an intensity of 168 *μ*mol/m^2^/s. Algal biomass was transferred to a mortar and pestle, and following flash freezing in liquid nitrogen, ground into a fine powder. While still frozen tissue was resuspended in working buffer: 10 mm potassium phosphate buffer (pH 7.0) containing 1 mm ascorbate, 20% w/v sorbitol, 1 mm EDTA, and 0.1% (v/v) phenylmethanesulfonylfluoride. The suspension was then passed through three layers of cheese cloth. To separate membrane and liquid fractions, the resulting cell lysate was spun at 4,000 × *g* for 20 min at 4 °C. The supernatant was collected, and the pellet fraction was resuspended in 1 mL of working buffer and both fractions were placed on ice. APX activity assays were conducted by measuring depletion of ascorbate through a change in absorbance at 300 nm using a photo spectrometer. Assay conditions were as follows: Reactions were carried out in 1 cm cuvettes containing 2 mL of reaction buffer, 50 mm potassium phosphate buffer containing 10 *µ*L of 100 mm ascorbate with 10 *µ*L of 20 mm H_2_O_2_ added to start the reaction. Initial readings were taken upon addition of the peroxide and subsequent readings were taken at 5-min intervals for a total of 20 min. The change in absorbance was used to calculate moles of ascorbate oxidized using Beer's law calculations (*ε* = 0.49 mm^−1^ cm^−1^). Results were normalized to protein content of samples with protein amount determined by Bradford assay, using Bio-Rad Protein Assay Solution (Bradford 1976).

### Dot blots and imaging

Constructs containing cDNA sequences for Chlamydomonas *LCI2* and *APX1* were ordered from Twist Bioscience and then inserted into the pDest14 overexpression plasmid before transformation of *E. coli* BL21 cell lines. For dual gene expression, tandem constructs were inserted into the pDest14 plasmid prior to transformation of BL21 cell lines. Cells were then induced by the addition of ITPG to 500 *µ*m once an OD of 0.5 was achieved at 17 °C overnight, shaking at 150 RPMs. Cells were then lysed using a French press @ 20,000 PSI and lysate was placed on Ice before centrifugation at 4500 × *g* for 30 min. The membrane pellet was then resuspended in BugBuster (Protein Extraction Reagent) containing Sigma complete Mini, EDTA-Free Protease Inhibitor Cocktail Ref #311836170001, at a concentration of 500:1 (initial culture volume:sample volume). Polyvinylidene difluoride (PVDF) membranes were then activated with methanol and 200 *µ*L of sample was placed in a dot on the membrane and allowed to bind for 5 min. Membranes were then blocked in powdered milk for 24 h at 4 °C before being washed three times with 0.1 m tris pH8. Samples were incubated with anti-His HRP conjugated antibodies or anti-HA antibodies (mouse) suspended in blocking buffer (25,000:1). For HA tags, following three more buffered washes, membranes were incubated for 1 h in a secondary antimouse HRP or alkaline phosphatase conjugated antibody. Membranes were then washed once with 0.1 m tris, 0.1% tween 20 and twice with 0.1 m tris prior to addition of reactive substrates. Imaging was done using a BioRad Chemi Doc MP imaging system. Expressed GFP was visualized using UV excitation channel while HRP was visualized using chemiluminescence channel. The visualization of HA tags was done using Alexa 488 nm excitation channel however due to overlap with GPF HA tag presence was confirmed with alkaline phosphatase staining.

### Statistical analyses

To determine statistical significance, samples were treated as independent groups and statistics were calculated using an unpaired, two-tailed *t*-test. Significance was noted by an asterisk with *P*-values of >0.05 and a double asterisk for samples with a *P*-value of >0.01.

### TBARS assay

TBARS (Thiobarbituric Acid Reactive Substances) assays were performed according to Li et al. (2012), with some modifications. Cultures were grown in 50 mL TAP medium or minimal medium using 250 mL Erlenmeyer Flasks at 25 °C under 12 h:12 h light:dark cycles. All reaction solutions were prepared immediately prior to performing the assays. Aliquots were sampled at reported time intervals and normalized to 1 OD prior to being subjected to centrifugation at 4,000 × *g* for 10 min in glass tubes using a bench top centrifuge. Supernatant was removed and TBA solution was added. The samples were mixed and then boiled for 1 h. Then samples were subjected to centrifugation as before and supernatant was transferred to 1 cm cuvettes to measure absorption at 532 nm.

### Accession numbers

Sequence data from this article for the LCI2/FAD4 locus can be found in the Phytozome data library (https://phytozome-next.jgi.doe.gov/info/CreinhardtiiCC_4532_v6_1) under the gene model identifier_ Cre16.g673001_4532 (v6.1).

## Supplementary Material

kiaf394_Supplementary_Data

## Data Availability

The data underlying this article are available in the article and in its online supplementary material.

## Supplementary data

The following materials are available in the online version of this article.

**Supplementary Figure S1.** Phylgenetic overview of the *LCI2/FAD4* locus.

**Supplementary Figure S2.** AlphaFold 3.0 structure predictions of various thylakoid asparatate peroxidases

**Supplementary Figure S3.** Expressed sequence tag (EST) coverage of *Cre16.g673001_4532.*

**Supplementary Figure S4.** Input DNA coverage and histone H3 lysine 4 (H3K3) methylation status at *Cre16.g673001_4532.*

**Supplementary Figure S5.**  *LCI2/FAD4* expression in response to CO_2_ limitation.

**Supplementary Figure S6.** Ascorbate peroxiodase activity of cytosolic and stromal fractions.

**Supplementary Figure S7.** LCI2 production in *E. coli.*

**Supplementary Table S1.** Organism and gene identifiers for APXs in **Supplementary Fig. S1**.

## Dive Curated Terms

The following phenotypic, genotypic, and functional terms are of significance to the work described in this paper:

lipid CHEBI: CHEBI:18059

APX1 Gramene: AT1G07890
APX1 Araport: AT1G07890

tAPX Gramene: AT1G77490
tAPX Araport: AT1G77490

FAD4 Gramene: AT4G27030
FAD4 Araport: AT4G27030

DCMU CHEBI: CHEBI:116509

NCBI Gramene: https://www.ncbi.nlm.nih.gov
NCBI Araport: https://www.ncbi.nlm.nih.gov
