# Supplementary material for: Low Carbon Inducible2/Fatty Acid Desaturase4 locus in C. reinhardtii directs plastid peroxidase location and trans fatty acid production
Source: Plant Physiol. 2025 Sep 3;199(1):kiaf394. doi: 10.1093/plphys/kiaf394 (PMC12456178; doi:10.1093/plphys/kiaf394)
Supplement: kiaf394_Supplementary_Data [file kiaf394_supplementary_data.zip › kiaf394_Supplementary_Data.pdf]

Supplemental Materials

The *LCI2/FAD4* locus in *C. reinhardtii* directs plastid peroxidase location and *trans* fatty acid production

Timothy J. Nicodemus, Stefan Schmollinger, John E. Froehlich, Daniela Strenkert, Barb B. Sears, and Christoph Benning

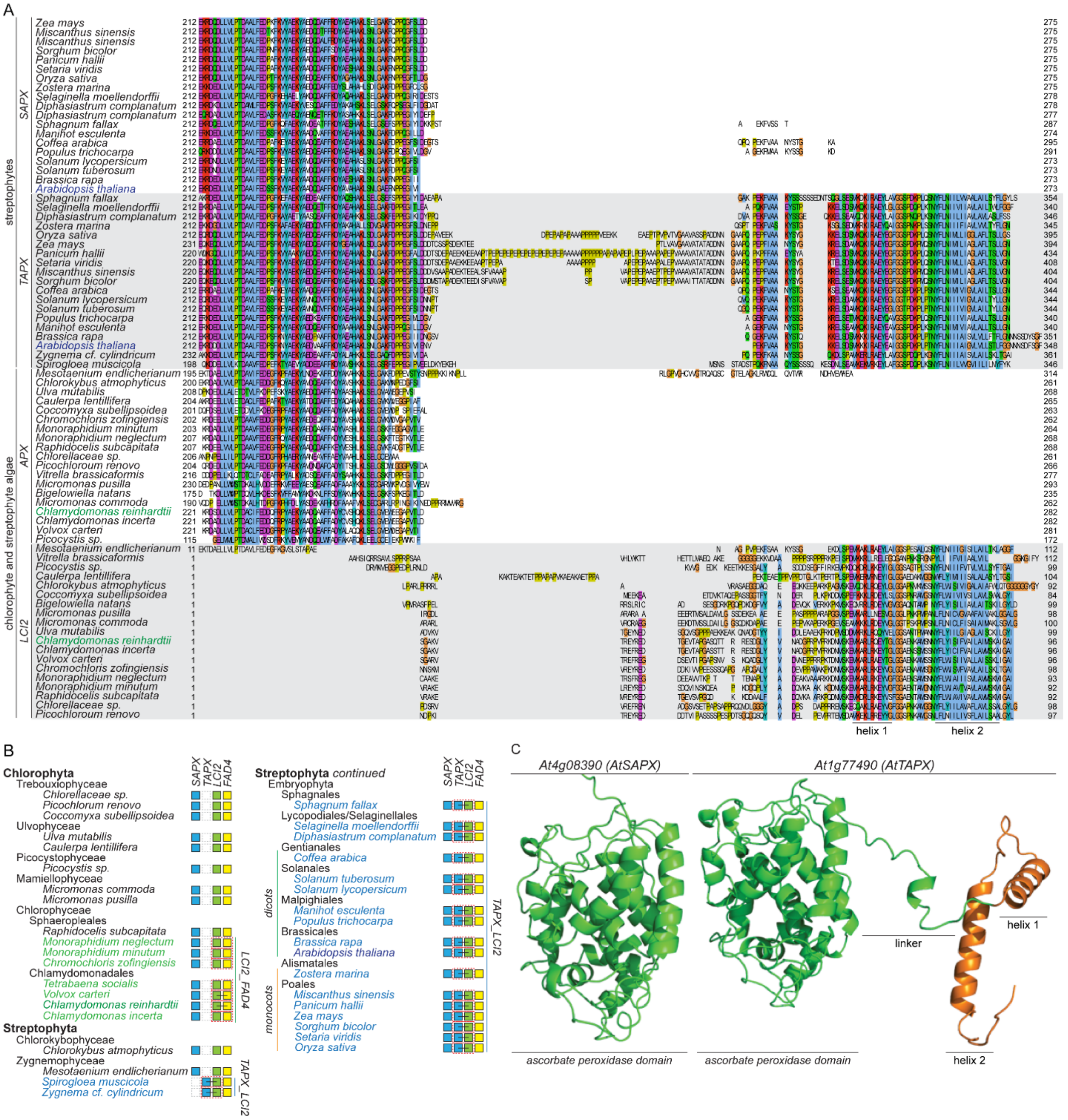

Supplemental Figure S1: Phylgenetic overview of the *LCI2/FAD4* locus. A, Aligment of C-terminal regions from stromal and thylakoid associated ascorbate peroxidases (APX) from different plants and green algae with LCI2. Sequences were retrieved from phytozome/phycocosm, chloroplast transit peptides were trimmed according to predictions by PredAlgo/TargetP and manual inspection of preliminary alignments, sequences are organized by alignment with ClustalO in Jalview and colored according to sequence conservation using the Clustal color scheme. B, Phylogenetic overview of the genomic association of LCI2 (green) with APX1 (SAPX, stromal APX, blue; TAPX thylakoid APX, white) FAD4 (yellow) in different plants and algae. Red outlined boxes indicate features from the same or directly adjacent loci, solid black lines between boxes indicated features of the same gene model. C, Alphafold 3 structure predictions of stromal and thylakoid-associated APX from *Arabidopsis thaliana* illustrating the domain composition at the C-terminus.

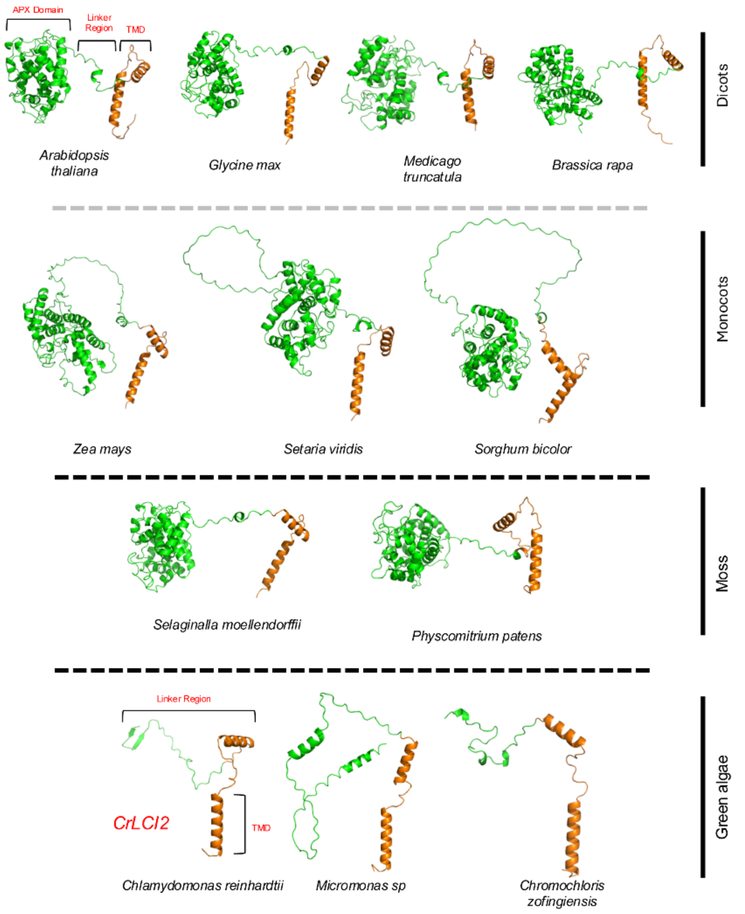

Supplemental Figure S2. **AlphaFold 3.0 structure predictions of various thylakoid asparatate peroxidases** (APX) from different plants and algae. Protein sequences were retrieved from Phytozome (v13, March 1, 2024; Goodstein et al., 2012) for the following species (genome version, identifier) [See Supplemental Figure 5] and were subsequently used to predict structures of various APXs: the dicots, *Arabidopsis thaliana* (TAIR11, AT1G77490.1), *Glycine max* (Wm82.a4.v1, 04G248300.1), *Medicago truncatula* (v4.0v1, 3g088160.1), *Brassica rapa* (FPsc v1.3, G03518.1); the moncots, *Zea mays* (V4, 00001d016802_T001), *Setaria viridis* (v2.1, 1G195800.1) and *Sorghum bicolor* (v3.1.1, 004G175500.1),; the mosses, *Physcomitrium patens* (v3.3, 3c1_40650V3.1), *Selaginalla moellendorffii* (v1.0, 85469), the green algae, *Chlamydomonas reinhardtii* (v6.1, Cre16.g673001_4532.4),*Micromonas sp.* RCC299 (v3.0, 108499) and *Chromochloris zofingiensis* (v5.2.3.2, 12g10220.t1). The best fit AlphaFold3.0 (Abramson et al., 2024) structure for each APX is presented. Green shading represents the ascorbate peroxidase domain (APX Domain) and the orange shading represents the Transmembrane Domain (TMD) which allows APX to be tethered to the thylakoid membrane. Note that for *Chlamydomonas reinhardtii* and other green algae only the C-terminal TMD is shown since their APX domains are independent soluble structures which may interact with the C-terminal domain under various conditions (See Model).

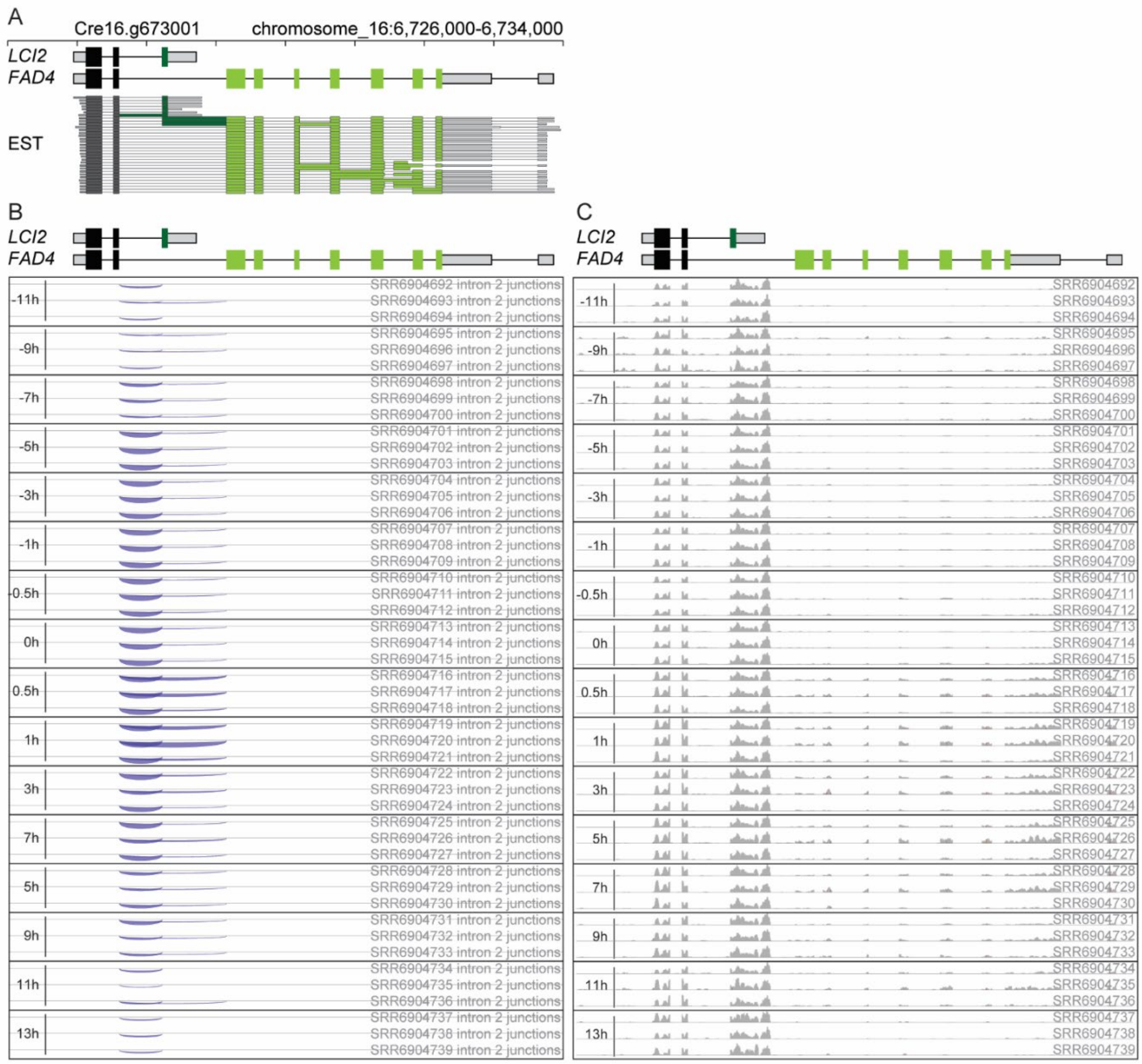

Supplemental Figure S3: Expressed sequence tag (EST) coverage of *Cre16.g673001_4532*, intron-spanning reads and read coverage throughout the day/night cycle (12h/12h). Data was remapped to version 6.1 of the Chlamydomonas genome (Craig et al., 2023). Highlighted are common (black), LCI2-unique (dark green) and FAD4-unique (light green) sequences, modified from transcripts *Cre16.g673001_4532.1* and *Cre16.g673001_4532.4.*

A, EST coverage of the *FAD4/LCI2* locus, adapted from (Goodstein et al., 2011, Asamizu et al., 2000, Asamizu et al., 2004).

B, abundance of intron_2-spanning reads, indicative of splicing events resulting either in *LCI2* or *FAD4* transcripts. Shown are three independent replicates for each timepoint along the diurnal cycle, adapted from (Strenkert et al., 2019).

C, read coverage along the *LCI2/FAD4* locus during the diurnal cycle. Shown are three independent replicates for each timepoint along the diurnal cycle, adapted from (Strenkert et al., 2019).

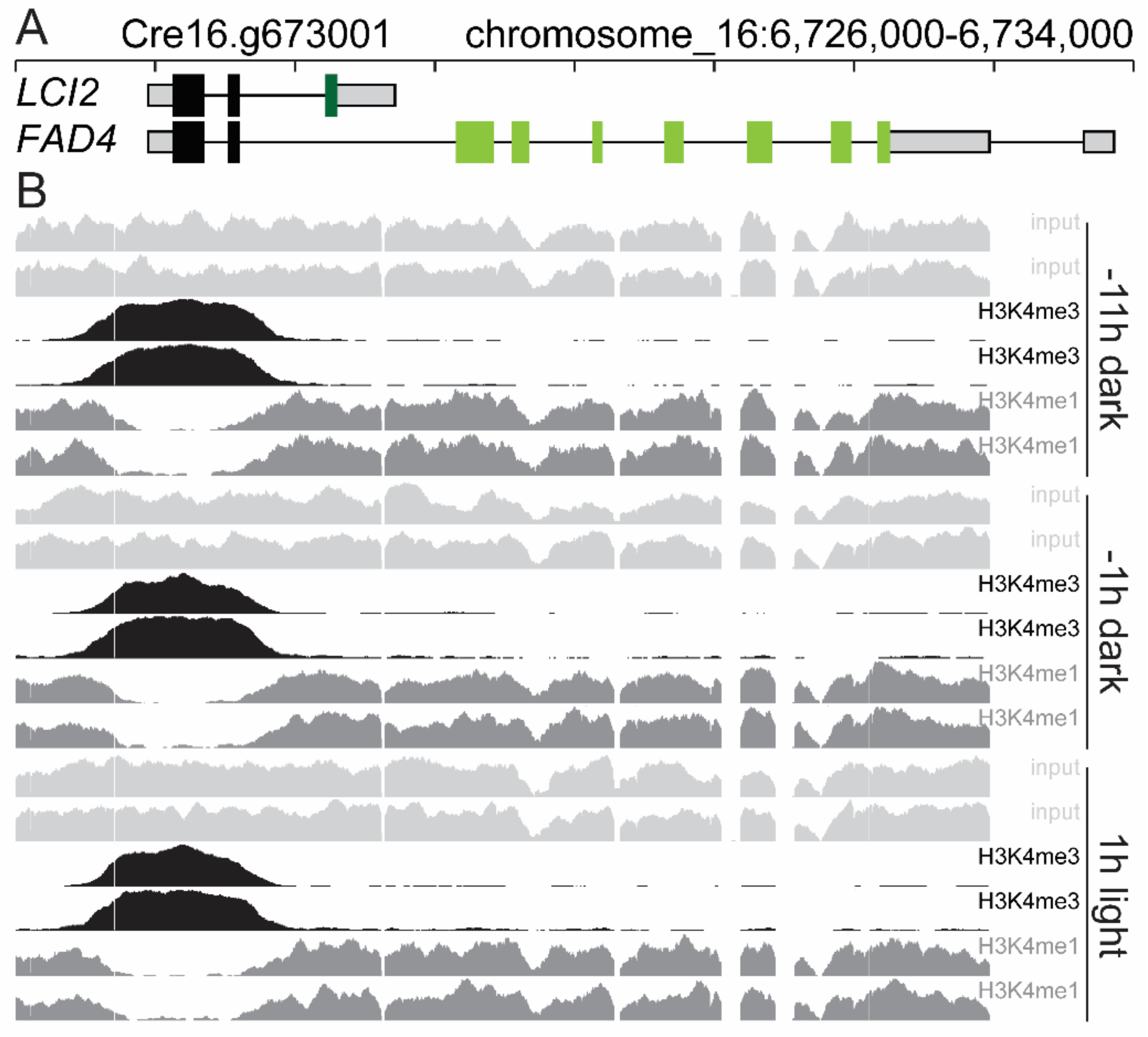

Supplemental Figure 4: Input DNA coverage and histone H3 lysine 4 (H3K3) methylation status at *Cre16.g673001_4532* throughout the day/night cycle (12h/12h).

A, two transcripts encoding for LCI2 and FAD4, respectively, originating from chromosome 16 (6,726,000-6,734,000) with common (black), LCI2-unique (dark green) and FAD4-unique (light green) exons (modified from transcripts *Cre16.g673001_4532.1-4,* Chlamydomonas genome v6.1 (Craig et al., 2023)).

B, read coverage showing input DNA, Histone H3, lysine 4 methylation status (H3K4me1/3), indicative of the presence of a transcriptional start site, at the *Cre16.g673001_4532* locus during a diurnal cycle (adapted from (Strenkert et al., 2022)). Shown are two independent replicates for each timepoint.

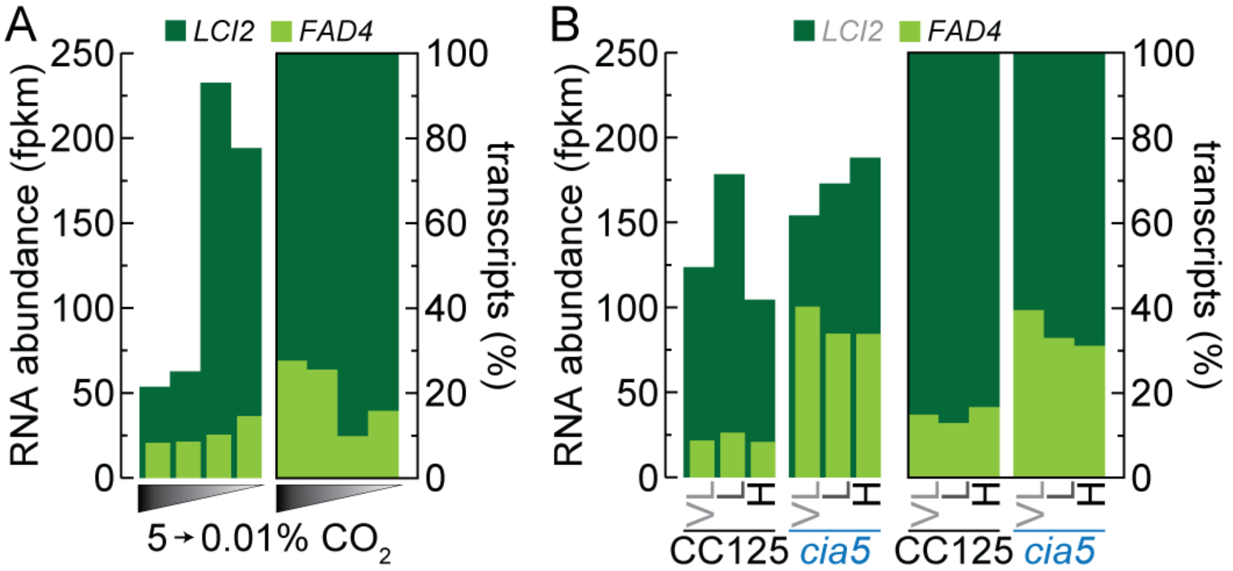

Supplemental Figure S5: *LCI2/FAD4* expression in response to CO_2 limitation.

A, transcript-specific expression (fpkm, fragments per kilobase of exon per million mapped reads, left scale) of *LCI2* (dark green fill) or *FAD4* (light green fill) and fraction of *FAD4* transcripts (%, right scale) among all transcripts originating from the locus in response to CO_2 deprivation in wild type Chlamydomonas cultures (CC-124, adapted from (Brueggeman et al., 2012)). Bars from left to right: 0, 30, 60, and 180 min after a reduction from 5 to 0.01% CO_2 supply. Shown is the average of two bioreactor runs.

B, transcript-specific expression (fpkm, left scale) of *LCI2* (dark green fill) or *FAD4* (light green fill) and fraction of *FAD4* transcripts (%, right scale) among all transcript originating from the locus in wild type (CC-125) and the *cia5* mutant, acclimated to different CO_2 environments (bars from left to right: cultures with 0.013% (very low, VL), 0.037% (low, L ~air levels) or 5% (high, H) CO_2 supply; adapted from (Fang et al., 2012)). Shown is the average of two cultures.

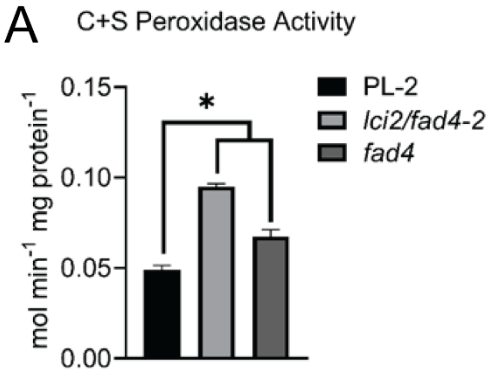

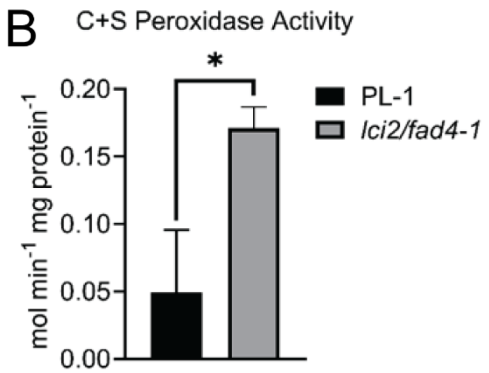

Supplemental Figure S6. Ascorbate Peroxidase Activity of cytosolic and stromal fractions. A, Enzymatic activity of cytosolic and stromal (C+S) fractions separated by centrifugation are shown in activity per mg of protein for CRISPR generated *lci2/fad4-2* and *fad4* mutants and their parental line (PL-2). B, enzymatic activity of stromal and cytosolic fractions separated by centrifugation are shown per mg of protein for the *lci2/fad4-1* mutant and its parental line (PL-1). SD is shown, sample size n=3; a 2- tailed t-test was used and significance was noted by an asterisk with p-values of >0.05.

GFP LCI2-HA

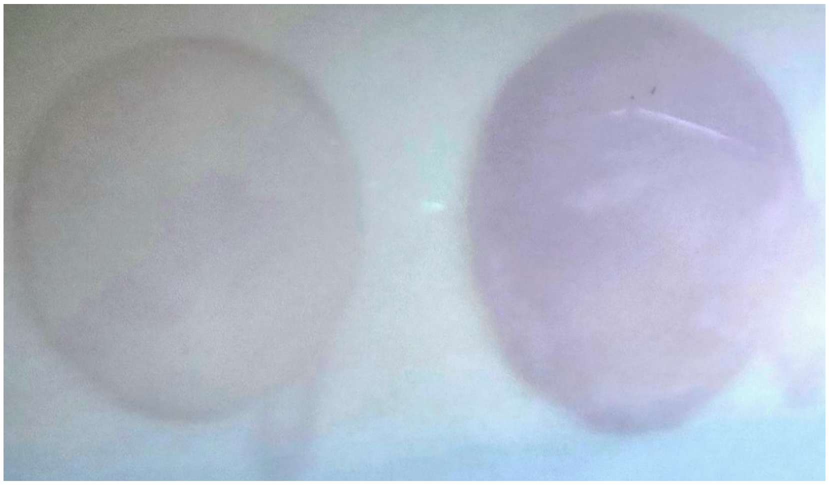

Supplemental Figure 7. LCI2 production in *E. coli.* Alkaline phosphatase staining of BL21 *E. coli* producing GFP (right) and HA tagged LCI2 (Left) following a dot blot on PVDF membrane post incubation with anti HA antibodies conjugated with alkaline phosphatase.

Supplemental Table S1. Organism and gene identifiers for APXs in Supplemental Fig. S1

organism	*LCI2*	*SATX*	*TATX*
Chlorellaceae sp	jgi|Trebou4966_1|40161	jgi|Trebou4966_1|21171	-
Picochloroum renovo	jgi|Picre1|27612	jgi|Picre1|34801	-
Coccomyxa subellipsoidea	jgi|Cosub3|1219330	jgi|Cosub3|1316442	-
Ulva mutabilis Føyn	jgi|Ulvmu1_1|11880	jgi|Ulvmu1_1|2888	-
Caulerpa lentillifera	jgi|Caulen1|89569	jgi|Caulen1|92658	-
Picocystis sp. ML	jgi|Pico_ML_1|52455	jgi|Pico_ML_1|52255	-
Micromonas commoda	jgi|MicpuN3v2|4762	jgi|MicpuN3v2|8497	-
Micromonas pusilla	jgi|MicpuC3v2|7022	jgi|MicpuC3v2|1135	-
Raphidocelis subcapitata NIES-35	jgi|Rapsub1_1|12432	jgi|Rapsub1_1|8745	-
Monoraphidium neglectum	jgi|Monneg1|7292	jgi|Monneg1|580	-
Monoraphidium minutum	jgi|Monmin1|266042	jgi|Monmin1|399201	-
Chromochloris zofingiensis	Cz12g10220.t1	Modified Cz06g15270.t1	-
Tetrabaena socialis	jgi|Tetso1_1|3896	jgi|Tetso1_1|14169	-
Volvox carteri	Vocar.0004s0445.1	Identified manually	-
Chlamydomonas reinhardtii	Cre16.g673001_4532.4	Cre02.g087700_4532.1	-
Chlamydomonas incerta	jgi|Chlin1|3941	jgi|Chlin1|3291	-
Chlorokybus atmophyticus	jgi|Chlat1_1|6991	jgi|Chlat1_1|2246	-
Mesotaenium endlicherianum	jgi|Mesen1_1|7557	jgi|Mesen1_1|3785	-
Bigelowiella natans	jgi|Bigna1|146434	jgi|Bigna1|51415	-
Vitrella brassicaformis	jgi|Vitbras1|23364	jgi|Vitbras1|18349	-
Spirogloea muscicola	-	-	jgi|Spimu1_1|8631
Zygnema cf. cylindricum	-	-	jgi|Zygcyl6981a_1|20641
Sphagnum fallax	-	Sphfalx05G037200.1	Sphfalx03G024900.1
Selaginella moellendorffii	-	156990	85469
Diphasiastrum complanatum	-	Dicom.10G026300.1 & Dicom.17G028700.1	Dicom.08G059700.1
Coffea arabica	-	evm.model.Scaffold_628.635	evm.model.Scaffold_506.82
Solanum lycopersicum	-	Solyc06g060260.3.1	Solyc11g018550.4.1
Solanum tuberosum	-	Soltu.DM.06G017340.1	Soltu.DM.11G011300.1
Manihot esculenta	-	Manes.18G083605.1	Manes.02G165100.1
Populus trichocarpa	-	Potri.002G081900.1	Potri.005G179200.2
Brassica rapa	-	Brara.I02406.1	Brara.G03518.1
Arabidopsis thaliana	-	AT4G08390.1	AT1G77490.1
Zostera marina	-	Zosma03g37080.1	Zosma01g42110.1
Miscanthus sinensis	-	Misin11G082400.1 & Misin12G084000.1	Misin08G162300.1
Panicum hallii	-	Pahal.7G153500.1	Pahal.1G265300.1
Zea mays	-	Zm00001d003643_T003	Zm00001d016802_T002
Sorghum bicolor	-	Sobic.006G084400.1	Sobic.004G175500.1
Setaria viridis	-	Sevir.7G110100.1	Sevir.1G195800.1
Oryza sativa	-	LOC_Os04g35520.1	LOC_Os02g34810.1

References cited in Supplemental Information

ASAMIZU, E., MIURA, K., KUCHO, K., INOUE, Y., FUKUZAWA, H., OHYAMA, K., NAKAMURA, Y. & TABATA, S. 2000. Generation of expressed sequence tags from low-CO2 and high-CO2 adapted cells of Chlamydomonas reinhardtii. *DNA Res,* 7**,** 305-7.

ASAMIZU, E., NAKAMURA, Y., MIURA, K., FUKUZAWA, H., FUJIWARA, S., HIRONO, M., IWAMOTO, K., MATSUDA, Y., MINAGAWA, J., SHIMOGAWARA, K., TAKAHASHI, Y. & TABATA, S. 2004. Establishment of publicly available cDNA material and information resource of Chlamydomonas reinhardtii (Chlorophyta) to facilitate gene function analysis. *Phycologia,* 43**,** 722-726.

BRUEGGEMAN, A. J., GANGADHARAIAH, D. S., CSERHATI, M. F., CASERO, D., WEEKS, D. P. & LADUNGA, I. 2012. Activation of the carbon concentrating mechanism by CO2 deprivation coincides with massive transcriptional restructuring in Chlamydomonas reinhardtii. *Plant Cell,* 24**,** 1860-75.

CRAIG, R. J., GALLAHER, S. D., SHU, S., SALOMÉ, P. A., JENKINS, J. W., BLABY-HAAS, C. E., PURVINE, S. O., O'DONNELL, S., BARRY, K., GRIMWOOD, J., STRENKERT, D., KROPAT, J., DAUM, C., YOSHINAGA, Y., GOODSTEIN, D. M., VALLON, O., SCHMUTZ, J. & MERCHANT, S. S. 2023. The Chlamydomonas Genome Project, version 6: Reference assemblies for mating-type plus and minus strains reveal extensive structural mutation in the laboratory. *The Plant Cell,* 35**,** 644-672.

FANG, W., SI, Y., DOUGLASS, S., CASERO, D., MERCHANT, S. S., PELLEGRINI, M., LADUNGA, I., LIU, P. & SPALDING, M. H. 2012. Transcriptome-wide changes in Chlamydomonas reinhardtii gene expression regulated by carbon dioxide and the CO2-concentrating mechanism regulator CIA5/CCM1. *Plant Cell,* 24**,** 1876-93.

GOODSTEIN, D. M., SHU, S., HOWSON, R., NEUPANE, R., HAYES, R. D., FAZO, J., MITROS, T., DIRKS, W., HELLSTEN, U., PUTNAM, N. & ROKHSAR, D. S. 2011. Phytozome: a comparative platform for green plant genomics. *Nuc Acid Res,* 40**,** D1178-D1186.

STRENKERT, D., SCHMOLLINGER, S., GALLAHER, S. D., SALOMÉ, P. A., PURVINE, S. O., NICORA, C. D., METTLER-ALTMANN, T., SOUBEYRAND, E., WEBER, A. P. M., LIPTON, M. S., BASSET, G. J. & MERCHANT, S. S. 2019. Multiomics resolution of molecular events during a day in the life of Chlamydomonas. *Proc Natl Acad Sci U S A,* 116**,** 2374-2383.

STRENKERT, D., YILDIRIM, A., YAN, J., YOSHINAGA, Y., PELLEGRINI, M., O'MALLEY, R. C., MERCHANT, S. S. & UMEN, J. G. 2022. The landscape of Chlamydomonas histone H3 lysine 4 methylation reveals both constant features and dynamic changes during the diurnal cycle. *The Plant Journal,* 112**,** 352-368.
