# Supplementary material for: Low Carbon Inducible2/Fatty Acid Desaturase4 locus in C. reinhardtii directs plastid peroxidase location and trans fatty acid production
Source: Plant Physiol. 2025 Sep 3;199(1):kiaf394. doi: 10.1093/plphys/kiaf394 (PMC12456178; doi:10.1093/plphys/kiaf394)
Supplement: kiaf394_Supplementary_Data [file PLPHYS-2025-1055 peer review report.pdf › PLPHYS-2025-1055 peer review report.pdf]

Email

**Manuscript #**	PP2024-RA-02292
**Title**	Alternative Splicing of LCI2/FAD4 in Chlamydomonas Links Chloroplast Lipid Metabolism and ROS Biochemistry
**Corresponding Author**	Dr. Christoph Benning (Michigan State University)
**Date:**	09-Sep-2024 08:22:21
**Last Sent:**	09-Sep-2024 08:22:21
**Created By:**	Alain Goossens
**From:**	pphys@msubmit.net
**To:**	benning@msu.edu
**Subject:**	PP2024-RA-02292: Decision Letter
**Email**	09-Sep-2024<br><br>Dr. Christoph Benning<br>Michigan State University<br>MSU-DOE Plant Research Laboratory<br>East Lansing, MI 48824-1319<br><br><br>RE: Alternative Splicing of LCI2/FAD4 in Chlamydomonas Links Chloroplast Lipid Metabolism and ROS Biochemistry<br><br>Dear Dr. Christoph Benning:<br><br>Thank you for giving us the opportunity to review your manuscript for publication in the Plant Physiology. The reviewers have found the manuscript of interest, but have raised a number of concerns that will require your attention. As these concerns are of some gravity and will entail substantial changes to the manuscript, you are likely to need additional time to address them. I must therefore reject the manuscript in its present form. Nonetheless, if you are able to address the points raised in review, I would welcome your resubmission of a manuscript that incorporates your responses.<br><br>Please note that any resubmission will be treated as a new entry to the system and is likely to go out for review.<br><br>If you do have any queries or concerns, you are welcome to contact me in the first instance.<br><br>Thank you again for allowing Plant Physiology to review your work. We look forward to hearing from you soon.<br><br>Sincerely,<br><br>Dr. Aruna Kilaru<br>Monitoring Editor, Plant Physiology<br><br>-- Reviewer comments:<br>Reviewer #1 (Comments for the Author):

The manuscript by Nicodemus and colleagues details an interesting investigation of the "unusual" FAD4 desaturase story from Bennning's group. In previous work they had identified FAD4 as a distant desaturase like gene responsible for the introduction of the delta 3 trans double bond into 16:0 in PG within the plastid. The lack of discernable phenotype in Arabidopsis FAD4 mutants motivated the current study the group investigated FAD4 function in Chlamydomonas because it is evolutionarily distant from Arabidopsis but contains FAD4. They discovered an unexpected finding in that an alternative message, encoding LCI2 is transcribed from the same promoter and contains the same first two exons. LCI2 is one of the Low CO2 Inducible genes, i.e., genes induced under carbon-limiting conditions. LCI2 has sequence similarity to acorbate peroxidase genes. Ascorbate peroxidases are redox proteins that protect cellular components from activated oxygen species.

The manuscript is very straightforward and the evidence for the transcription of both genes is solid. Significant differences in the differential expression of the sliced transcripts during day night cycles is demonstrated. The search for distinct functions is achieved by disruption the FAD4 or both genes, however, no differences in growth were observed over the 12 days. I was not clear why panel B lacks a trace for a FAD4 mutant.
In Figure 3. This data confirms the identity of the Chlamydomonas FAD4 because the mutant lacks the 16:1D3 t. I am confused by their claim of an increase in de novo fatty acid syntheses can be drawn from data presented in mole %. It seems such a claim would be better evidenced by absolute data rather than percentage. Also, a simple, classical acetate tracer feeding experiment might be more appropriate to support this. A minor point, but I found it difficult to read the histograms because they had thick lines and similar shading. This could be fixed to make it easier to interpret.
Figure 4 demonstrates the ascorbate peroxidase activity of LCI2, however the observation that LCI2 mutants had compensatory increase in peroxidase activity is intriguing and would be interesting to follow up as to the basis for this activity.
T+M and C + S need to be defined in the figure legend.
The authors are refreshingly open that the implications of co-regulation of FAD4 and LCI3 are presently unknown. While they show that LCI2 is conserved across a number of algae, it would be interesting to know if they are more widely present in nature.
The authors describe a possible scenario in which 16:1D3t might be oxidized by ROS and this might explain a coupling between peroxidase and desaturase function to renew levels of the 16:1D3t.
This raises two issues. First it would be helpful for the authors to create a cartoon to summarize this hypothesis. Second, is there any data on the relative susceptibility of oxidation of 16:1D3t relative to other plastid fatty acids? If 16:1D3t were particularly sensitive to such oxidation, it would provide support for this suggestion.
Very minor wording suggestion: In the introduction, the phrase "and remain longer active providing", might be better stated as "remain active longer"

Reviewer #2 (Comments for the Author):

In this work, the authors have studied the LCI2/FAD4 locus in Chlamydomonas. Through a series of results, the authors show that an alternative splicing event generates two distinct enzymes, LCI2, a protein with ascorbate peroxidase activity and the desaturase FAD4. The authors have analyzed expression data and obtained mutants to characterize this locus, proposing that there could be a link between this alternative splicing event, the production of 16:1-trans to stabilize photosynthetic membrane proteins through FAD4 activity and eliminate ROS through the action of LCI2 ascorbate peroxidase. The hypothesis proposed by the authors is very interesting and some of the results point to that direction. However, in my opinion there is still more experiments to do in order to obtain enough data to support

their hypothesis.

Here are my detailed comments to their results:

1.- The gene structure of this LCI2/FAD4 locus in Chlamydomonas is very particular. It does not occur in higher plants, where the FAD4 locus is well characterized in Arabidopsis and many other plants. Therefore, it could be inferred that this gene structure is an exception of algae. Authors should provide in the introduction section information concerning whether this gene structure is also present in the genomes of other algae. This is relevant to know if it is an exception of Chlamydomonas and also very important, to understand whether the proposed mechanism is present only in Chlamydomonas or it is more general in other algae, which would give the results more relevancy.

2.- It is clear from the results that it is an alternative splicing event (ASE) that generates the two different enzymes. Therefore, it could be expected that both transcripts share the same TSP, as shown by the authors. I would recommend to perform specific primer extension experiments with non-radiactive modified nucleotides to determine if when the ASE occurs, the TSP is really the same. The histone analysis is OK, but it is bioinformatics, and there could be differences in the TSP that could be relevant. I say this because it is hard to understand how the same transcription factors are going to control the expression of two genes with so different activities. What kind of cis regulatory elements are detectable in the promoter? Can they be related with the activities of both enzymes?

3.- It is also clear from the results presented in the paper that the ASE is activated by light, particularly in the first hour upon illumination. In the night, or upon different carbon availability, the LCI2 part of the gene is much more expressed than the FAD4 one, indicating that the ASE is off. However, given that the hypothesis from the authors is that the LCI2/FAD4 locus might play a role in the elimination of ROS species and protecting or stabilizing membrane lipids proximal to photosynthetic complexes, I wonder what could be the results under high light experiments. Under such conditions, both genes would be forced. It would be very interesting to see how high light affects the ASE and, for example, if differences in the different mutants tested appear in growth, ROS enzymes activity or even fatty acid compositions. These results might help to reinforce the hypothesis proposed by the authors as presented in the conclusion with more experimental evidences.

4.- To continue with this, I would strongly recommend to analyze lipid peroxidation in the WT, the mutants and the different growing conditions, including the high light experiments. This may help to connect the expression data, the occurrence of the ASE, and effective lipid protection/stabilization.

5.- Finally the authors should validate the expression data from the RNA-Seq analysis by qPCR, choosing primers specific of the LCI2, FAD4 in different parts of the gene to confirm its whole transcription.

Reviewer #3 (Comments for the Author):

The manuscript by Nicodemus et al. reports the characterization, in the green alga Chlamydomonas reinhardtii, of two partly overlapping genes (LCI2 and FAD4) driven by the same promoter but encoding different products as a result of differential splicing. LCI2 encodes a product inducible by exposure to low ambient levels of CO_2. FAD4 encodes a fatty acid desaturase that generates a 16-carbon trans double bond-containing fatty acid found exclusively in phosphatidylglycerol of chloroplast membranes. The authors speculate

that the unique organization and expression of these genes provide a connection between chloroplast lipid metabolism and responses to reactive oxygen species (ROS).

The reanalysis of RNA-seq and EST data from several published genome-wide studies supports the production of two alternative transcripts (encoding either LCI2 or FAD4) from the C. reinhardtii Cre16.g673001_4532 locus (Fig. 1A/B and Supplemental Fig. 3). The reanalysis of the distribution of trimethylated and monomethylated lysine 4 on histone H3 suggested that both mRNAs share a single transcription start site (Fig. 1C and Supplemental Fig. 2). The abundance of both transcripts peaked early in the day (0.5 to 1 h after turning the lights on) and the relative proportion of the FAD4 transcript (relative to the total transcripts produced from the locus) was higher during the day (Fig. 1D/E and Supplemental Fig. 3). The relative abundance of the 16:1Δ3trans fatty acid along the diurnal cycle was also higher during the day (Fig. 1F).

The authors generated two edited mutants with premature stop codons (by using CRISPR/Cpf1 with donor oligodeoxynucleotides as repair templates) to block the production of both LCI2 and FAD4 (mutant lci2fad4-2) or only FAD4 (mutant fad4) (Fig. 2A). They also examined an insertional mutant (lci2fad4-1) obtained from the Chlamydomonas library (Fig. 2A). However, the mutants did not show any growth defect under photoautotrophic conditions when compared to their parental strains (Fig. 2B/C). The 16:1Δ3trans fatty acid was not detected in any of the mutants with disrupted production of the longer protein (Fig. 3), corroborating that the longer spliced transcript encodes a FAD4 desaturase.

The LCI2 predicted protein sequence showed similarity to canonical thylakoid-bound ascorbate peroxidases (Supplemental Fig. 5). Interestingly, peroxidase activity associated with membrane fractions (cellular and thylakoid fractions) was reduced in the mutants defective in LCI2 but not in the fad4 mutant (Fig. 4A/B). Conversely, an increase in peroxidase activity in the cytosolic/stromal fractions was observed in all the lci2 mutants, potentially a compensatory response to a deficiency in thylakoid peroxidase activity (Fig. 4C/D). Overexpression of recombinant His-tagged LCI2 in E. coli resulted in a 2-fold increase in ascorbate peroxidase activity in cell extracts, relative to a control overexpressing GFP (Fig. 4E).

In summary, the authors demonstrated the production of two differentially spliced transcripts from the C. reinhardtii Cre16.g673001_4532 locus. The mutant analysis convincingly supports that the longer transcript encodes a FAD4 desaturase, responsible for the production of the 16:1Δ3trans fatty acid. However, it is not entirely clear that LCI2 encodes a thylakoid-bound ascorbate peroxidase (please see main comments below). Moreover, there is no experimental support for the proposed link, based on the co-regulation of LCI2 and FAD4, between chloroplast lipid metabolism and REDOX biochemistry. Overall, the manuscript is well-written and easy to follow.

Main comments:
1. Since none of the mutants showed defective growth under normal photoautotrophic conditions, the biological role(s) of LCI2 and FAD4 is not clear. The authors postulate that the co-regulation of LCI2 and FAD4 implies a link between chloroplast lipid metabolism and responses to ROS (i.e., REDOX biochemistry), as emphasized in the title of the manuscript. However, in the absence of direct experimental evidence, this assertion remains highly speculative. The authors could perhaps explore whether the mutants are more sensitive to certain abiotic stresses expected to increase ROS production associated with photosynthetic activity (such as exposure to high light, low levels of DCMU, etc.). Are LCI2, FAD4 or both required for tolerance to these abiotic stresses? Are they required for the maintenance of photosynthetic performance under these stress conditions?

2. Canonical thylakoid-bound ascorbate peroxidases have a catalytic domain of ~250 amino acids and their full length is around 400 amino acids. LCI2 is a much shorter protein (131 aa) and the mature protein may be even shorter assuming that it has a cleavable chloroplast transit peptide. Moreover, LCI2 shows greater similarity to the carboxy-terminal region of canonical thylakoid-bound ascorbate peroxidases (Supplemental Fig. 5). However, this region is outside of the catalytic domain and may code for a transmembrane domain. Given these considerations, it does not seem certain that LCI2 is an ascorbate peroxidase. Does purified His-tagged LCI2 (for instance by metal ion affinity chromatography) still show ascorbate peroxidase activity? Does structural homology modeling (for instance using Swiss Model) reveal greater similarity to the catalytic domain of ascorbate peroxidases?

3. This reviewer is not familiar with ascorbate peroxidase assays in crude cell extracts. Assuming that the assays are specific, the results could also be explained if LCI2 encodes an activator of canonical ascorbate peroxidases (but in itself is not an ascorbate peroxidase). Could the addition of His-tagged LCI2 enhance the activity of ascorbate peroxidases (if a suitable enzyme is commercially available)?

4. Page 7, lines 225-230. It is not clear why an increase in the abundance of 16:0 fatty acids is interpreted as evidence of an increase in de novo fatty acid synthesis. Could this be the fatty acid that is not converted to 16:1Δ3trans (and remains as 16:0)?

5. Several sentences refer to Fig. 1 (or a panel of Fig. 1) that was not included in the manuscript. I am assuming this was a diagram of the locus with the target sites for CRISPR/Cpf1 and the changes introduced by editing. Please check pages 6 (line 206), 7 (line 238), 9 (line 313) and 10 (lines 351 and 368).

Minor comments:
1. Page 6, line 185. I think that this sentence refers to Supplemental Fig. 3.

2. Page 6, lines 188-189. This sentence is not clear. Does "in" need to be deleted?

3. Could you explain under Materials and Methods how the abundance of the differentially spliced transcripts was examined? Did you use exclusively unique junction-spanning reads?

4. I know that this is described under Material and Methods but it would be helpful to the readers to add a sentence in the legend to the figures mentioning the statistical analysis and the significance of the asterisks (and the meaning of ND in Fig. 4B).

5. Are you sure that the reference Pham et al. (2022b) is correct (page 14, lines 558-563)?

Dear Editor,

We have carefully considered the very helpful feedback provided by the reviewers, rewritten large sections of the paper, changed the title and implemented the following changes to address their concerns. We believe this is a much better paper now. Our responses are in blue.

Reviewer 1 Comments:

"The manuscript is very straightforward and the evidence for the transcription of both genes is solid. Significant differences in the differential expression of the sliced transcripts during day night cycles is demonstrated. The search for distinct functions is achieved by disruption the FAD4 or both genes, however, no differences in growth were observed over the 12 days. I was not clear why panel B lacks a trace for a FAD4 mutant."

Additional growth curves in TAP media have been added (FIG 5) demonstrating differences in growth. It should be noted that a mutant only disrupted in *FAD4* is not available in the CLip library where the *lci2/fad4-1* mutant was obtained from.

"In Figure 3. This data confirms the identity of the Chlamydomonas FAD4 because the mutant lacks the 16:1D3 t. I am confused by their claim of an increase in de novo fatty acid syntheses can be drawn from data presented in mole %. It seems such a claim would be better evidenced by absolute data rather than percentage. Also, a simple, classical acetate tracer feeding experiment might be more appropriate to support this. A minor point, but I found it difficult to read the histograms because they had thick lines and similar shading. This could be fixed to make it easier to interpret."

We included the absolute amounts in Fig 3. now. We did not follow up further on detailing the more far-reaching effects on lipid biosynthesis as we think this is a distraction for the flow of the current paper and will follow up on this issue in a subsequent study.

"Figure 4 demonstrates the ascorbate peroxidase activity of LCI2, however the observation that LCI2 mutants had compensatory increase in peroxidase activity is intriguing and would be interesting to follow up as to the basis for this activity.
T+M and C + S need to be defined in the figure legend."

We are now discussing that LCI2 is a membrane anchor recruiting APX to the membrane, something we missed before, and which provides an explanation for the apparent compensatory effect of soluble APX. An experiment has been added (Fig. 4) which addresses the concern of LCI2 compensatory response vs localization which examines the LCI2 role as a membrane anchor that recruits/stabilizes ascorbate peroxidases at the membrane. This experiment addresses the localization of APX, a soluble plastid ascorbate peroxidase, in the presence and absence of LCI2.

"The authors are refreshingly open that the implications of co-regulation of FAD4 and LCI3 are presently unknown. While they show that LCI2 is conserved across a number of algae, it would be interesting to know if they are more widely present in nature."

We now took a deeper dive into the evolution of this locus and added/revised Supplemental Figures 1 and 2 and added an in-depth section at the beginning of the results to demonstrate gene location and structure across algal species and terrestrial plants as well as characterization of terrestrial plants evolutionary development of dedicated thylakoid ascorbate peroxidases.

"The authors describe a possible scenario in which 16:1D3t might be oxidized by ROS and this might explain a coupling between peroxidase and desaturase function to renew levels of the 16:1D3t.
This raises two issues. First it would be helpful for the authors to create a cartoon to summarize this hypothesis. Second, is there any data on the relative susceptibility of oxidation of 16:1D3t relative to other plastid fatty acids? If 16:1D3t were particularly sensitive to such oxidation, it would provide support for this suggestion."

We toned down this aspect of the paper and refocused it on the role of LCI2 as a membrane anchor for APX. However, we added new data to show the loss of LCI2/FAD4 increases ROS damage to membranes (Fig. 6), which does speak to this point and is discussed accordingly now.

"Very minor wording suggestion: In the introduction, the phrase "and remain longer active providing", might be better stated as "remain active longer"

We made the change.

Reviewer 2 Comments:

"In this work, the authors have studied the LCI2/FAD4 locus in Chlamydomonas. Through a series of results, the authors show that an alternative splicing event generates two distinct enzymes, LCI2, a protein with ascorbate peroxidase activity and the desaturase FAD4. The authors have analyzed expression data and obtained mutants to characterize this locus, proposing that there could be a link between this alternative splicing event, the production of 16:1-trans to stabilize photosynthetic membrane proteins through FAD4 activity and eliminate ROS through the action of LCI2 ascorbate peroxidase. The hypothesis proposed by the authors is very interesting and some of the results point to that direction. However, in my opinion there is still more experiments to do in order to obtain enough data to support their hypothesis.

Here are my detailed comments to their results:

1.- The gene structure of this LCI2/FAD4 locus in Chlamydomonas is very particular. It does not occur in higher plants, where the FAD4 locus is well characterized in Arabidopsis and many other plants. Therefore, it could be inferred that this gene structure is an exception of algae. Authors should provide in the introduction section information concerning whether this gene structure is also present in the genomes of other algae. This is relevant to know if it is an exception of Chlamydomonas and also very important, to understand whether the proposed mechanism is present only in Chlamydomonas or it is more general in other algae, which would give the results

As mentioned above, to address this point we now took a deeper dive into the evolution of this locus and added/revised Supplemental Figures 1 and 2 and added an in-depth section at the beginning of the results to demonstrate gene location and structure across algal species and terrestrial plants as well as characterization of terrestrial plants evolutionary development of dedicated thylakoid ascorbate peroxidases. We strongly agree that this adds more substance to our discovery of this interesting locus.

2.- It is clear from the results that it is an alternative splicing event (ASE) that generates the two different enzymes. Therefore, it could be expected that both transcripts share the same TSP, as shown by the authors. I would recommend to perform specific primer extension experiments with non-radiactive modified nucleotides to determine if when the ASE occurs, the TSP is really the same. The histone analysis is OK, but it is bioinformatics, and there could be differences in the TSP that could be relevant. I say this because it is hard to understand how the same transcription factors are going to control the expression of two genes with so different activities. What kind of cis regulatory elements are detectable in the promoter? Can they be related with the activities of both enzymes?

The data we present are not just bioinformatics data but based on extensive RNAseq data that were previously published and reanalyzed by us. Based on these data even in the absence of a more detailed experimentation as proposed, for which we are unfortunately not setup, the most likely scenario is that the transcript expression for both variants (LCI2/FAD4) is driven by the same promoter elements and transcription factors, which is supported by the findings shown in Figure 1C and Supplemental Figures 3 and 4, that demonstrate that *LCI2/FAD4* generally show similar expression dynamics. The splicing event however appears to occur in 3 discrete steps, creating more *FAD4*-ASE during daytime (especially early in the day), less so in the extended night and even less so during cell division. This most likely is regulated at the pre-mRNA level by RNA binding proteins post-transcriptionally, having different affinities or activities in the light, or in response to CO2 changes. Based on our data including new data on ROS activity in the mutants (Fig. 6) the co expression of FAD4 and the LCI2 anchor for the membrane recruitment of APX possibly to the same location as FAD4 makes sense and supports our hypothesis.

3.- It is also clear from the results presented in the paper that the ASE is activated by light, particularly in the first hour upon illumination. In the night, or upon different carbon availability, the LCI2 part of the gene is much more expressed than the FAD4 one, indicating that the ASE is off. However, given that the hypothesis from the authors is that the LCI2/FAD4 locus might play a role in the elimination of ROS species and protecting or stabilizing membrane lipids proximal to photosynthetic complexes, I wonder what could be the results under high light experiments. Under such conditions, both genes would be forced. It would be very interesting to see how high light affects the ASE and, for example, if differences in the different mutants tested appear in growth, ROS enzymes activity or even fatty acid compositions. These results might help to reinforce the hypothesis proposed by the authors as presented in the conclusion with more experimental evidences.

Instead of applying high light, we investigated growth in the presence of different concentrations of DCMU to slow down or block PSII at ambient growth light, as also proposed by another reviewer to

examine the effects of photosynthetic ROS production. These new results are now shown in Figure 5 and discussed accordingly.

4.- To continue with this, I would strongly recommend to analyze lipid peroxidation in the WT, the mutants and the different growing conditions, including the high light experiments. This may help to connect the expression data, the occurrence of the ASE, and effective lipid protection/stabilization.

We did perform these experiments, and they are now shown in Figure 6 and discussed accordingly.

5.- Finally the authors should validate the expression data from the RNA-Seq analysis by qPCR, choosing primers specific of the LCI2, FAD4 in different parts of the gene to confirm its whole transcription."

To address this point, qPCR was performed to confirm expression of 2 different transcripts as shown in Figure 2A. Additionally, this experiment also revealed *lci2/fad4* mutants obtained from the CLip library exhibited small amounts of LCI2 transcripts explaining some of our observations as being due to a leaky mutation in this case.

Reviewer 3 Comments:

"The manuscript by Nicodemus et al. reports the characterization, in the green alga Chlamydomonas reinhardtii, of two partly overlapping genes (LCI2 and FAD4) driven by the same promoter but encoding different products as a result of differential splicing. LCI2 encodes a product inducible by exposure to low ambient levels of CO2. FAD4 encodes a fatty acid desaturase that generates a 16-carbon trans double bond-containing fatty acid found exclusively in phosphatidylglycerol of chloroplast membranes. The authors speculate that the unique organization and expression of these genes provide a connection between chloroplast lipid metabolism and responses to reactive oxygen species (ROS).

The reanalysis of RNA-seq and EST data from several published genome-wide studies supports the production of two alternative transcripts (encoding either LCI2 or FAD4) from the C. reinhardtii Cre16.g673001_4532 locus (Fig. 1A/B and Supplemental Fig. 3). The reanalysis of the distribution of trimethylated and monomethylated lysine 4 on histone H3 suggested that both mRNAs share a single transcription start site (Fig. 1C and Supplemental Fig. 2). The abundance of both transcripts peaked early in the day (0.5 to 1 h after turning the lights on) and the relative proportion of the FAD4 transcript (relative to the total transcripts produced from the locus) was higher during the day (Fig. 1D/E and Supplemental Fig. 3). The relative abundance of the 16:1Δ3trans fatty acid along the diurnal cycle was also higher during the day (Fig. 1F).

The authors generated two edited mutants with premature stop codons (by using CRISPR/Cpf1 with donor oligodeoxynucleotides as repair templates) to block the production of both LCI2 and FAD4 (mutant lci2fad4-2) or only FAD4 (mutant fad4) (Fig. 2A). They also examined an insertional mutant (lci2fad4-1) obtained from the Chlamydomonas library (Fig. 2A). However, the mutants did not show any growth defect under photoautotrophic conditions when compared to their parental strains (Fig. 2B/C). The 16:1Δ3trans fatty acid was not detected in any of the mutants with disrupted production of the longer protein (Fig. 3), corroborating that the longer spliced transcript encodes a

FAD4 desaturase.

The LCI2 predicted protein sequence showed similarity to canonical thylakoid-bound ascorbate peroxidases (Supplemental Fig. 5). Interestingly, peroxidase activity associated with membrane fractions (cellular and thylakoid fractions) was reduced in the mutants defective in LCI2 but not in the fad4 mutant (Fig. 4A/B). Conversely, an increase in peroxidase activity in the cytosolic/stromal fractions was observed in all the lci2 mutants, potentially a compensatory response to a deficiency in thylakoid peroxidase activity (Fig. 4C/D). Overexpression of recombinant His-tagged LCI2 in E. coli resulted in a 2-fold increase in ascorbate peroxidase activity in cell extracts, relative to a control overexpressing GFP (Fig. 4E).

In summary, the authors demonstrated the production of two differentially spliced transcripts from the C. reinhardtii Cre16.g673001_4532 locus. The mutant analysis convincingly supports that the longer transcript encodes a FAD4 desaturase, responsible for the production of the 16:1Δ3trans fatty acid. However, it is not entirely clear that LCI2 encodes a thylakoid-bound ascorbate peroxidase (please see main comments below). Moreover, there is no experimental support for the proposed link, based on the co-regulation of LCI2 and FAD4, between chloroplast lipid metabolism and REDOX biochemistry. Overall, the manuscript is well-written and easy to follow.

Main comments:
"1. Since none of the mutants showed defective growth under normal photoautotrophic conditions, the biological role(s) of LCI2 and FAD4 is not clear. The authors postulate that the co-regulation of LCI2 and FAD4 implies a link between chloroplast lipid metabolism and responses to ROS (i.e., REDOX biochemistry), as emphasized in the title of the manuscript. However, in the absence of direct experimental evidence, this assertion remains highly speculative. The authors could perhaps explore whether the mutants are more sensitive to certain abiotic stresses expected to increase ROS production associated with photosynthetic activity (such as exposure to high light, low levels of DCMU, etc.). Are LCI2, FAD4 or both required for tolerance to these abiotic stresses? Are they required for the maintenance of photosynthetic performance under these stress conditions?"

We added growth curves in TAP medium with different concentrations of DCMU present that show a difference in growth between the mutants and the parental lines (Fig. 5). Additionally, assays to detect lipid peroxidation prior to and during DCMU treatment were carried out using TBAR analysis also mentioned in response to similar suggestions made by reviewer 2.

2. Canonical thylakoid-bound ascorbate peroxidases have a catalytic domain of ~250 amino acids and their full length is around 400 amino acids. LCI2 is a much shorter protein (131 aa) and the mature protein may be even shorter assuming that it has a cleavable chloroplast transit peptide. Moreover, LCI2 shows greater similarity to the carboxy-terminal region of canonical thylakoid-bound ascorbate peroxidases (Supplemental Fig. 5). However, this region is outside of the catalytic domain and may code for a transmembrane domain. Given these considerations, it does not seem certain that LCI2 is an ascorbate peroxidase. Does purified His-tagged LCI2 (for instance by metal ion affinity chromatography) still show ascorbate peroxidase activity? Does structural homology

modeling (for instance using Swiss Model) reveal greater similarity to the catalytic domain of ascorbate peroxidases?

This was an excellent point that we admittingly missed and which changed the entire story. Thank you for pointing this out. Additional bioinformatic and structural analysis of LCI2 and chloroplast APX enzymes were performed. Analysis revealed LCI2 exhibited a similar structure as the membrane anchoring portions of higher plant thylakoid bound ascorbate peroxidases but does not conmtain an active APX site. Further modeling of protein-protein interactions between APX1 and LCI2 suggested LCI2's involvement in recruitment and stabilization of active APX1. This prompted us to investigate the localization of APX 1 in an LCI2 dependent manner (Fig 4). The "compensatory" effect of soluble APX in the mutants can now be understood as lack of membrane recruitment of APX in the mutants. We changed the text and the title accordingly.

3. This reviewer is not familiar with ascorbate peroxidase assays in crude cell extracts. Assuming that the assays are specific, the results could also be explained if LCI2 encodes an activator of canonical ascorbate peroxidases (but in itself is not an ascorbate peroxidase). Could the addition of His-tagged LCI2 enhance the activity of ascorbate peroxidases (if a suitable enzyme is commercially available)?

Efforts to purify LCI2 and study it directly were unsuccessful as the protein ended up in inclusion bodies common with membrane proteins. However, as mentioned above already, additional modeling and APX/LCI2 localization experiments were carried out to address this concern.

4. Page 7, lines 225-230. It is not clear why an increase in the abundance of 16:0 fatty acids is interpreted as evidence of an increase in de novo fatty acid synthesis. Could this be the fatty acid that is not converted to 16:1Δ3trans (and remains as 16:0)?

As also addressed for reviewer 1, absolute amounts were added, but we did tone down the effects on wider lipid metabolism as we feel this will distract from the main message of the current paper. We believe this topic warrants further investigation, but as a separate story.

5. Several sentences refer to Fig. 1 (or a panel of Fig. 1) that was not included in the manuscript. I am assuming this was a diagram of the locus with the target sites for CRISPR/Cpf1 and the changes introduced by editing. Please check pages 6 (line 206), 7 (line 238), 9 (line 313) and 10 (lines 351 and 368).

We fixed this issue in the revision. Sorry about the confusion this caused.

Minor comments:
1. Page 6, line 185. I think that this sentence refers to Supplemental Fig. 3.

Sentence was amended.

2. Page 6, lines 188-189. This sentence is not clear. Does "in" need to be deleted?

Sentence was amended.

3. Could you explain under Materials and Methods how the abundance of the differentially spliced transcripts was examined? Did you use exclusively unique junction-spanning reads?

We did add more detail to the methods. To address the question here directly: The *abundance of the different splice forms was determined using only reads that exclusively mapped to LCI2/FAD4-specific regions. For this purpose, the LCI2/FAD4 locus on chromosome 16 was changed in the .gff3 feature file of the Chlamydomonas genome (v6.1) that was used for differential expression analysis with cuffdiff from the Cufflinks package (v2.2.1)* . The modified locus only contained 2 mRNAs (not 4) originating from the locus, which only included regions that are exclusive to either LCI2 (last exon + 3'UTR) or FAD4 (last 7 exons + 3'UTR). Separately, reads directly spanning the splice junction of LCI2 or FAD4, respectively, were isolated using the view function of SAMtools#.*

**Differential analysis of gene regulation at transcript resolution with RNA-seq Cole Trapnell, David Hendrickson, Martin Sauvageau, Loyal Goff, John L. Rinn, Lior Pachter Nature Biotechnology, 2012, doi:10.1038/nbt.2450*

#Li H, Handsaker B, Wysoker A, Fennell T, Ruan J, Homer N, Marth G, Abecasis G, Durbin R, and 1000 Genome Project Data Processing Subgroup, The Sequence alignment/map (SAM) format and SAMtools, Bioinformatics (2009) 25(16) 2078-9

4. I know that this is described under Material and Methods but it would be helpful to the readers to add a sentence in the legend to the figures mentioning the statistical analysis and the significance of the asterisks (and the meaning of ND in Fig. 4B).

Figure legends were amended.

5. Are you sure that the reference Pham et al. (2022b) is correct (page 14, lines 558-563)?”

We had some double refs in the MS. The current Pham 2022 ref is the correct citation referencing the cell wall reduced arginine auxotroph lines generated via CRISPR and double stranded repair.

Date: Jun 30, 2025
To: "Christoph Benning" benning@msu.edu
cc: algoo@psb.vib-ugent.be
From: "Plant Physiology" pphys-submissions@aspb.org
Subject: PLPHYS-2025-1055 Decision Letter

JOURNAL: Plant Physiology
MSID: PLPHYS-2025-1055
MS TITLE: Alternative Splicing of LCI2/FAD4 in Chlamydomonas allows for coordinated membrane recruitment of APX and FAD4
CA: Dr. Christoph Benning

Dear Dr. Benning,

We are pleased to accept your manuscript for publication in Plant Physiology. This acceptance is contingent on revision based on the comments of our reviewers.

Please highlight all changes and include a detailed annotation to changes to the text, with line numbers, and noting your responses to the comments.

If you cannot return the revised manuscript within 4 weeks of receipt, please let us know. Otherwise, we will assume that you have elected not to revise the manuscript and are withdrawing it.

Thank you for allowing us to review your work. We look forward to hearing from you soon.

Sincerely,

Aruna Kilaru
Monitoring Editor
Plant Physiology

Reviewers' comments:
Reviewer 1: I have carefully read the revised version of the paper by Nicodemus et al. I have also considered the answers to my review points of the previous version. In my opinion the authors have answered to all of my points in a proper way. This new version has considerably improved its quality. and some of the new data and figures are very interesting. It is a very nice work.

Reviewer 3: The manuscript by Nicodemus et al. investigates the transcriptional regulation of an interesting locus in Chlamydomonas encoding both LCI2 and FAD4. This is a revised manuscript for which I was not reviewer on the initial submission. The authors found that LCI2 and FAD4 are expressed from the same locus through alternative splicing regulated in part by carbon availability and also time in daylight. They confirmed that FAD4 is responsible for the production of 16:1delta3trans PG in Chlamydomonas and discovered that LCI2 serves as a membrane anchor to recruit peroxidase activity through the comparison of single fad4 mutant and lci2/fad4 double mutants.

There is a question as to whether the results, as currently reported, fully support the conclusion that co-expression of LCI2 and FAD4 has functional relevance (especially in light of Fig5A,B, please see my comment below for line 312). The title (coordinated recruitment) and text could be revised to ensure the conclusions tightly align to the findings currently included. This study reports interesting results, I provide below some suggestions to strengthen the methodology, and to increase the clarity in the interpretation of the results.

Major comments

Line 283, consider including the analysis of chloroplast-localized APX expression to ensure that lack of peroxidase activity in lci2 (Fig4A,B) is not due to less APX protein accumulation, alternatively include this possibility in the text. It is unlikely considering the higher activity in C+S, but that could be driven by upregulation of cytosolic APX.
Line 288, explain the choice of APX1 specifically and provide more introduction on the number and localization of APXs.
Line 312, consider analyzing the transcript accumulation in these conditions (as well as mixotrophic no add for control), as the expression might be changed e.g. LCI2 might not be reduced by 50% in the -1 allele due to ROS signaling to provide possible explanation for the lack of growth impairment in this mutant. Assuming LCI2 is back to wild type level here to explain the rescued growth, how to explain the lack of effect from the fad4-1 mutation, if indeed FAD4 has an impact on growth and ROS production?
Figure 4A,B and 5C,D: please include an explanation for the large differences among the strains (peroxidase activity) 0.008 in PL-1 vs 0.03 in PL-2 (especially as they are similar for C+S) and (TBARS) 0.15 vs 0.3 at day 1 for example.
Figure 4: the title needs to be changed as LCI2 itself doesn't display APX activity.
Figure 2B,C and Figure 6: include the light intensity used for growth and explain the differences in growth rate between the conditions. Describe the TBAR experiment in the method section.

Minor comments

Line 153, consider including alignment of FAD4 from different species to clarify whether FAD4 is membrane anchored in plants for example. I put it here as a minor comment, but it has 'major' implications in discussing a potential coordinated recruitment at the membrane.
Line 223, refer to Fig1E.
Line 235, considering referring to the mutants as lci2-1/fad4-1 and lci2-2/fad4-2 so it's clear that lci2 is impacted differently in these two alleles. Or fad4/lci2-1 and fad4/lci2-2 may be more intuitive as fad4 is impacted similarly in both, but not lci2.
Line 304, please avoid using "higher" because it infers superiority between species, that complexity increased with the evolution of eukaryotes, which is not always the case. Vascular or land plants can be used instead if wanting to put emphasis in contrast with algae for example.
Figure 4A,B: define "T+M".
Figure 4C,D: scale bars and labels to describe briefly the experiment would orient the reader.
Figure 5 and 6 could be condensed together (showing 1 or 5 μM DCMU is sufficient as the response is similar). Including labels on the graphs describing the conditions e.g. "+DCMU" would help the reader.
Suppl. 1B: Correct SATX and TATX to SAPX and TAPX. Define the red dashed box and line. There are several typos, please correct e.g. retrieved, phylogenetic, different, supplemental in Table 1 title.
Supplemental table 2 is missing.
Describe supplemental in order, 4 is before 3 in the text or reorder supplemental figures.

In compliance with data protection regulations, you may request that we remove your personal registration details at any time. (Use the following URL: https://www.editorialmanager.com/plphys/login.asp?a=r). Please contact the publication office if you have any questions.

Response to Reviewer Click here to access/download;Response to Reviewer;ResponseLetter.pdf

Response Letter

Reviewer 1: I have carefully read the revised version of the paper by Nicodemus et al. I have also considered the answers to my review points of the previous version. In my opinion the authors have answered to all of my points in a proper way. This new version has considerably improved its quality. and some of the new data and figures are very interesting. It is a very nice work.

Thank you for the positive comments.

There was no Reviewer 2 comment

Reviewer 3: The manuscript by Nicodemus et al. investigates the transcriptional regulation of an interesting locus in Chlamydomonas encoding both LCI2 and FAD4. This is a revised manuscript for which I was not reviewer on the initial submission. The authors found that LCI2 and FAD4 are expressed from the same locus through alternative splicing regulated in part by carbon availability and also time in daylight. They confirmed that FAD4 is responsible for the production of 16:1delta3trans PG in Chlamydomonas and discovered that LCI2 serves as a membrane anchor to recruit peroxidase activity through the comparison of single fad4 mutant and lci2/fad4 double mutants.

This is a new, fourth, reviewer of this manuscript who comes up with new concerns. We are responding to the best of our ability and have made edits where reasonable.

There is a question as to whether the results, as currently reported, fully support the conclusion that co-expression of LCI2 and FAD4 has functional relevance (especially in light of Fig5A,B, please see my comment below for line 312). The title (coordinated recruitment) and text could be revised to ensure the conclusions tightly align to the findings currently included. This study reports interesting results, I provide below some suggestions to strengthen the methodology, and to increase the clarity in the interpretation of the results.

We do feel the results are fully supported by the conclusions but as a compromise, we changed the title to: "The *LCI2/FAD4* locus in *C. reinhardtii* directs plastid peroxidase location and *trans* fatty acid production". And we made edits in response to the reviewer's comments as outlined below.

"Major comments

Line 283, consider including the analysis of chloroplast-localized APX expression to ensure that lack of peroxidase activity in lci2 (Fig4A,B) is not due to less APX protein accumulation,

alternatively include this possibility in the text. It is unlikely considering the higher activity in C+S, but that could be driven by upregulation of cytosolic APX.

We added a sentence as we cannot completely rule out this possibility without further analysis.

Line 288, explain the choice of APX1 specifically and provide more introduction on the number and localization of APXs.

We added our logic for choosing APX1 and two references on which our choice was based.

Line 312, consider analyzing the transcript accumulation in these conditions (as well as mixotrophic no add for control), as the expression might be changed e.g. LCI2 might not be reduced by 50% in the -1 allele due to ROS signaling to provide possible explanation for the lack of growth impairment in this mutant. Assuming LCI2 is back to wild type level here to explain the rescued growth, how to explain the lack of effect from the fad4-1 mutation, if indeed FAD4 has an impact on growth and ROS production?

We are not sure we understand this point. The fad4-1 mutation cannot be separated from the lci2/fad4-1 mutation. The lci2/fad4-1 mutation has a slight growth defect, much less than the lci2/fad4-2 growth defect consistent with the less severe reduction in transcript levels (Figure 2A).

Figure 4A,B and 5C,D: please include an explanation for the large differences among the strains (peroxidase activity) 0.008 in PL-1 vs 0.03 in PL-2 (especially as they are similar for C+S) and (TBARS) 0.15 vs 0.3 at day 1 for example.

Figure 5CD does not show peroxidase activity. Differences in the peroxidase activity of the two parental strains shown in Figure 4 AB are most certainly due to the differences in genetic background. PL1 is a cell wall mutant line while, PL2 is a cell-walled line. One simple explanation is that relative more protein can be extracted from PL1 than PL2 under our conditions which provides the denominator for the activity number given.

Figure 4: the title needs to be changed as LCI2 itself doesn't display APX activity.

Thanks, good catch. This was left from the previous version. We modified the title of Figure 4.

Figure 2B,C and Figure 6: include the light intensity used for growth and explain the differences in growth rate between the conditions.

Light intensities were the same for all experiments as described under growth conditions. Figure 2 is autotrophic growth, Figure 6 mixotrophic growth explaining the difference. The media used for each experiment are given in the two figure legends.

Describe the TBAR experiment in the method section.

Thanks, we somehow missed that. It is now added.

Minor comments

Line 153, consider including alignment of FAD4 from different species to clarify whether FAD4 is membrane anchored in plants for example. I put it here as a minor comment, but it has 'major' implications in discussing a potential coordinated recruitment at the membrane.

The membrane association of FAD4 and its orthologs is well established in the desaturase field. We have published a structure prediction of Arabidopsis FAD4 with the four transmembrane helixes in https://doi.org/10.1111/tpj.14657, Figure S3. We do not believe an additional FAD4 sequence line up will add more information, but we are now noting the transmembrane domains of the Chlamydomonas ortholog in the text where the reviewer indicated.

Line 223, refer to Fig1E.

Done

Line 235, considering referring to the mutants as lci2-1/fad4-1 and lci2-2/fad4-2 so it's clear that lci2 is impacted differently in these two alleles. Or fad4/lci2-1 and fad4/lci2-2 may be more intuitive as fad4 is impacted similarly in both, but not lci2.

We understand the point and have spent considerable time arguing among us about the best way to describe the allele situation of this complex locus. In the end, we agreed that our current allele designation is consistent with accepted nomenclature, unambiguous, and we decided to keep it.

Line 304, please avoid using "higher" because it infers superiority between species, that complexity increased with the evolution of eukaryotes, which is not always the case. Vascular or land plants can be used instead if wanting to put emphasis in contrast with algae for example.

Agreed. We changed it to seed plants.

Figure 4A,B: define "T+M".

Done

Figure 4C,D: scale bars and labels to describe briefly the experiment would orient the reader.

We added a description of the approximate diameter to the figure legend. We do not understand the question for additional labels. The spots are labeled.

Figure 5 and 6 could be condensed together (showing 1 or 5 μM DCMU is sufficient as the response is similar). Including labels on the graphs describing the conditions e.g. "+DCMU" would help the reader.

We disagree as the experiments described in the two figures are independent and can't be directly compared. We also believe the labeling, color coding, and descriptions in the figure legends are clear and unambiguous.

Suppl. 1B: Correct SATX and TATX to SAPX and TAPX. Define the red dashed box and line. There are several typos, please correct e.g. retrieved, phylogenetic, different, supplemental in Table 1 title.

Thanks. The mistakes were corrected.

Supplemental table 2 is missing.

There is no Supplemental Table 2. We searched the document and did not find a reference to it.

Describe supplemental in order, 4 is before 3 in the text or reorder supplemental figures.

Thanks. We reversed the order of the two supplemental figures.

Date: Jul 16, 2025
To: "Christoph Benning" benning@msu.edu

From: "Plant Physiology" pphys-submissions@aspb.org
Subject: PLPHYS-2025-1055R1 Decision Letter

JOURNAL: Plant Physiology
MSID: PLPHYS-2025-1055R1
MS TITLE: The LCI2/FAD4 locus in C. reinhardtii directs plastid peroxidase location and trans fatty acid production
CA: Dr. Christoph Benning

Dear Dr. Benning,

I am pleased to inform you that your revised manuscript "The LCI2/FAD4 locus in C. reinhardtii directs plastid peroxidase location and trans fatty acid production" has been accepted for publication in Plant Physiology.

Your manuscript will be checked for consistency with journal formatting and image standards by one of our Science Editors, who will contact you shortly. They may have a number of queries and suggestions for you. Please keep in mind that they work to increase the online visibility of your article and its attraction for readers from the widest of backgrounds. You will have the opportunity to review any revisions, and I hope you will work constructively with them to get the most out of your research. You will also have the opportunity to review all changes when you receive the author proofs for your article.

Once validated for publication, your article will be sent to a Science Editor for a final review of your manuscript and source files. After your final files are uploaded, your article will appear in the next available issue and the online Preview version of your article will be posted after your signed Copyright and Checklist forms and final source files are received.

Please be sure to include the following in your manuscript file:
Harvard style citations
List of all author contributions and funding information at the beginning of manuscript after the titles and author list.
List all supplemental materials associated with the article, with titles, at the end of the ms, before the references and acknowledgments section.
Complete Figure Legends for all figures appearing in the article listed before the Literature Cited.

LICENSE INFORMATION: Shortly after your paper is sent to production, you will receive an email from our publisher's author support team, SciPris, with information on article processing charges, as well as the types of Open Access license you would be able to purchase. The accepted version of your manuscript will not appear on our Advance Articles page until the license is selected and signed, so please look out for an email from SciPris. For more information see the Article Publication Fees section of our author guidelines: https://academic.oup.com/plcell/pages/General-Instructions

Finally, we encourage your submission of artwork for the journal cover. If there is an image or illustration related to your paper that you would like to have considered, please email it in .pdf or .tif format to clowe@aspb.org. An image title, brief, 2-3 sentence description of the image, and the name of the person credited for the image are also encouraged. If your image is selected to appear on the online cover of the journal, you will be contacted with the scheduled issue date and further information.

Thank you and congratulations on your Plant Physiology paper!

Sincerely,
Dr Alain Goossens
Monitoring Editor
Plant Physiology

=====
FIRST AUTHOR PROFILES
=====

We recognize the hard work that goes into being First Author of a Plant Physiology paper by publishing a "First Author Feature" for each First Author alongside your paper.

If the first author(s) would like to submit information for a feature on Facebook and Twitter, fill out the form by clicking on the link below:

Plant Physiology First Author Feature Form: https://forms.office.com/r/ZBCGZfhm8F

If you cannot access the form through the link above, please contact Rachel at rbelsky@aspb.org.

To upload an image of yourself to accompany your profile, please put your photo in the correct journal folder at the link below. You must label your image: Author First Name Last Name Article Title.

First Author Profile Image Dropbox: https://www.dropbox.com/scl/fo/xz029p7qjr2jml2rp2kmq/h?dl=0&rlkey=6fkxcaqmxx35ap152b2osy1fz

=====
IMPORTANT REMINDER: PEER REVIEW REPORTS
=====

If you opted to publish a peer review report along with your article during the original submission process, it will be prepared by the editorial staff and publicly posted with your manuscript, inside the zip file that contains any other supplemental material. As a reminder, the peer review report is a public record of all comments from editors and reviewers, as well as your prior responses, as you received them in the decision letters for each draft of your manuscript. If you agreed to publish this report and have changed your mind, or are not sure if you selected this option, please contact the editorial office as soon as possible before signing the license agreement from our publisher.

Comments from the Editors and Reviewers:

In compliance with data protection regulations, you may request that we remove your personal registration details at any time. (Use the following URL: https://www.editorialmanager.com/plphys/login.asp?a=r). Please contact the publication office if you have any questions.
